# The Role of Electrolyte Composition in Enabling Li Metal‐Iron Fluoride Full‐Cell Batteries

**DOI:** 10.1002/advs.202105803

**Published:** 2022-02-24

**Authors:** Bryan R. Wygant, Laura C. Merrill, Katharine L. Harrison, A. Alec Talin, David S. Ashby, Timothy N. Lambert

**Affiliations:** ^1^ Department of Photovoltaics and Materials Technology Sandia National Laboratories Albuquerque NM 87185 USA; ^2^ Department of Nanoscale Sciences Sandia National Laboratories Albuquerque NM 87185 USA; ^3^ Department of Quantum and Electronic Materials Sandia National Laboratories Livermore CA 94550 USA

**Keywords:** conversion electrodes, electrolyte compatibility, iron fluoride cathode, Li metal anodes, lithium batteries

## Abstract

FeF_3_ conversion cathodes, paired with Li metal, are promising for use in next‐generation secondary batteries and offer a remarkable theoretical energy density of 1947 Wh kg^−1^ compared to 690 Wh kg^−1^ for LiNi_0.5_Mn_1.5_O_4_; however, many successful studies on FeF_3_ cathodes are performed in cells with a large (>90‐fold) excess of Li that disguises the effects of tested variables on the anode and decreases the practical energy density of the battery. Herein, it is demonstrated that for full‐cell compatibility, the electrolyte must produce both a protective solid‐electrolyte interphase and cathode‐electrolyte interphase and that an electrolyte composed of 1:1.3:3 (m/m) LiFSI, 1,2‐dimethoxyethane, and 1,1,2,2‐tetrafluoroethyl‐2,2,3,3‐tetrafluoropropyl ether fulfills both these requirements. This work demonstrates the importance of verifying electrode level solutions on the full‐cell level when developing new battery chemistries and represents the first full cell demonstration of a Li/FeF_3_ cell, with both limited Li and high capacity FeF_3_ utilization.

## Graphical abstract

FeF_3_‐Li metal batteries have a high theoretical capacity, but their performance is typically limited by insufficient anode‐ and cathode‐electrolyte compatibility. Careful selection of an appropriate electrolyte system is needed to allow for stable cycling. It is found that an electrolyte containing a fluorinated ether (1,1,2,2‐tetrafluoroethyl‐2,2,3,3‐tetrafluoropropyl ether/1,2‐dimethoxyethane) is compatible with both the FeF_3_ cathode and the Li metal anode.

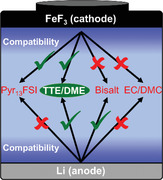

## 1 Introduction

As the demand for energy storage continues to grow, conversion cathodes for secondary Li‐ion and Li metal batteries are among the most promising avenues for next‐generation rechargeable batteries. Because they can access more than one electron of charge per redox metal center with reasonable stability, conversion cathodes have significantly higher capacities than more traditional intercalation cathodes like LiNi_1.5_Mn_0.5_O_4_ (690 Wh kg^−1^) or LiFePO_4_ (90–160 Wh kg^−1^) when paired with high capacity Li metal anodes. Among conversion cathodes, FeF_3_ is one of the most promising materials due to its high theoretical capacity (712 mAh g^−1^), high theoretical cell voltage versus Li metal (3.82 V),^[^ ^1^ ^]^ and the large abundance of Fe in the Earth's crust (6.3% by mass) relative to other common cathode metals like Ni (0.009% by mass) or Co (0.003% by mass).^[^ ^2^ ^]^ Paired with a Li metal anode, FeF_3_ represents an exciting path for secondary batteries, with high energy density (1947 Wh kg^−1^, 6855 Wh L^−1^).^[^ ^3^ ^]^

FeF_3_ conversion cathodes suffer from several intrinsic properties, including the low electronic conductivity of the material before and after discharge,^[^ ^4^, ^5^ ^]^ the slow reaction kinetics and relative irreversibility of the conversion reaction,^[^ ^3^ ^]^ and the solubility of Fe in common battery electrolytes.^[^ ^6^ ^]^ However, recent work on FeF_3_ material processing and especially electrolyte choice has shown that these challenges can be partially overcome. For example, both cyclic carbonates (e.g., ethylene carbonate/propylene carbonate) and ionic liquid (IL) electrolytes (e.g., *N*‐methyl‐*N*‐propyl pyrrolidinium bis(fluorosulfonylimide), or Pyr_13_FSI) have been shown to produce beneficial cathode electrolyte interphase (CEI) layers on FeF_3_ and FeF_2_ electrodes (referred to collectively herein as FeF*_x_*) that improve the stability and performance of the cathodes.^[^ ^7^, ^8^ ^]^ Although these and other studies^[^ ^9^, ^10^ ^]^ clearly demonstrate the promise of improving the performance of FeF*_x_* cathodes through electrolyte engineering, it is necessary to also consider the compatibility of the electrolyte with the anode to achieve a truly full‐cell level (i.e., potentially, practically relevant) solution.^[^ ^11^ ^]^

To address this issue, we have examined a series of three electrolytes optimized for either FeF*_x_* materials or Li metal, as well as a commercial carbonate‐based electrolyte (**Table** **1**), to better elucidate the role of electrolyte choice for a full cell FeF_3_/Li battery. First, we tested an electrolyte containing 1 m LiFSI in Pyr_13_FSI IL solvent (hereafter referred to as Pyr_13_FSI) based on its previously promising performance with FeF_2_ cathodes,^[^ ^8^ ^]^ although its suitability for Li metal remains questionable.^[^ ^12^ ^]^ In addition to potential anode incompatibility, the IL is incompatible with common polypropylene separators,^[^ ^13^ ^]^ requiring instead thicker glass fiber separators, making it less attractive for use in commercial cells. Based on recent work demonstrating Li/electrolyte compatibility, we next chose two promising electrolytes for use with Li anodes. The first is an electrolyte containing high concentrations of both LiFSI and LiTFSI in a 50:50 (v/v) mixture of 1,3‐dioxoloane (DOL) and 1,2‐dimethoxyethane (DME),hereafter referred to as bisalt, and the second is a localized high‐concentration electrolyte (LHCE) containing LiFSI and a highly fluorinated ether (1,1,2,2‐tetrafluoroethyl‐2,2,3,3‐tetrafluoropropyl ether, TTE) in DME (hereafter referred to as TTE/DME); both show high Li cycling efficiency and good self‐discharge behavior.^[^ ^14^, ^15^, ^16^ ^]^ The TTE/DME electrolyte also showed a wide voltage stability window up to 4.5 V versus Li, making it a strong choice for high voltage cycling. Given the apparent compatibility of the LiFSI‐based IL electrolyte with FeF*_x_* cathodes,^[^ ^4^ ^]^ we hypothesized that the TTE/DME could also be a suitable full cell solution for FeF_3_/Li batteries. Finally, as a control, we tested a commercially available electrolyte containing LiPF_6_ in a mixture of ethylene carbonate (EC) and dimethyl carbonate (DMC), which is not expected to be compatible with either Li or FeF_3_.^[^ ^8^, ^15^ ^]^

**Table 1:** Abbreviated label, salt concentration, and solvent composition of electrolyte solutions used in all coin cells tested

Electrolyte label	Salt and concentration	Solvent and composition
Pyr_13_FSI	1 m LiFSI	*n‐*Propyl‐*n*‐methylpyrrolidinium bis(fluorosulfonyl)imide
TTE/DME	1.73 m LiFSI	1,1,2,2‐Tetrafluoroethyl 2,2,3‐tetrafluoropropylrther+1,2‐dimethoxyethane, 3.65:1(v/v)
Bisalt	2 m LiFSI + 1 m LiTFSI	1,3‐Dioxoloane+1,2‐dimethoxyethane, 1:1(v/v)
EC/DMC	1 m LiPF	Ethylene carbonate + dimethyl carbonate, 1:1(v/v)

After choosing these electrolytes, we used a variety of electrochemical, physical, and chemical characterization techniques to study the compatibility of each electrolyte with both a nanocomposite FeF_3_/C cathode and a Li metal anode, and therefore determine the suitability of each electrolyte for use in a full cell battery. We began by conducting galvanostatic battery testing of FeF_3_ cathodes paired with excess Li, comparing the coulombic efficiency (CE) and capacity/retention to determine what impact each electrolyte had without limitations from the Li anode. We then conducted CE analysis of thin Li anodes in each electrolyte using a Cu counter electrode to study the efficiency of Li stripping and plating in each electrolyte in the absence of FeF_3_. Together, these electrochemical experiments allowed us to determine that the TTE/DME electrolyte was the only electrolyte compatible with both the FeF_3_ and Li metal. Following this determination, we used both scanning electron microscopy (SEM) and scanning transmission electron microscopy (STEM) to study the electrodes before and after testing and study the relationship between electrolyte‐related physical changes and the earlier electrochemical performance. We likewise used X‐ray photoelectron spectroscopy (XPS) to study chemical differences in the CEI and solid electrolyte interphase (SEI) of cathodes and anodes, respectively, to determine what impact electrolyte choice has on the chemical composition of these layers. We found that electrolyte choice directly impacts the chemical composition of both the CEI and SEI, and that the TTE/DME electrolyte is the only electrolyte that concurrently creates both a favorable CEI and SEI. Finally, using the information gained above, we galvanostatically tested full cell batteries built using a limited‐Li anode (20 µm thick). Our results showed that only the cells containing TTE/DME demonstrated good capacity retention and CE behavior. Thus, TTE/DME is a promising full cell solution for Li metal anode/FeF_3_ cathode batteries because of its ability to produce protective surface layers on both Li metal and FeF_3_; the other electrolytes only create such a layer on a single electrode and are therefore less compatible with full cell batteries.

## 2 Results and Discussion

### 2.1 Electrochemical FeF_3_/Electrolyte Compatibility Testing

**Figure 1:**
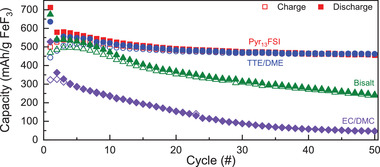
Charge/discharge capacity of FeF_3_/Li cells in various electrolyte solutions at C/20 versus a 750 µm Li anode (*n* = 1). Both Pyr_13_FSI and TTE/DME show similar capacity retention over 50 cycles.

First, to determine the compatibility of each electrolyte chemistry with the cathode material, we built FeF_3_‐limited full‐cells using FeF_3_ cathodes and a 750 µm‐thick Li metal anode (90‐fold excess capacity, Table S1, Supporting Information). Cycling the cells at C/20, we find relatively high discharge capacities near 550 mAh g^−1^ (77% of the theoretical capacity of FeF_3_) are maintained for the first five cycles in the Pyr_13_FSI, TTE/DME, and bisalt electrolytes (**Figure** **1**). In contrast to this high performance in LiFSI‐ and LiTFSI‐based electrolytes, FeF_3_ cathodes cycling in the 1 m LiPF_6_ in the 50:50 ethylene carbonate/dimethyl carbonate (EC/DMC) (v/v) control electrolyte (hereafter referred to as EC/DMC) showed capacities closer to 325 mAh g^−1^. Average discharge capacities based on triplicate measurements of cells using these chemistries support these results and are available in Figures S1–S5 (Supporting Information). The EC/DMC control cells then rapidly lose most of the capacity within 30 cycles, likely due to Fe dissolution aided by the hexafluorophosphate anion.^[^ ^6^ ^]^ A similar, but less severe, loss of capacity is also observed in the bisalt cells after ten cycles. Given that the electrolyte is reported to cycle Li with high efficiency,^[^ ^16^, ^17^ ^]^ this suggests that the capacity loss is likely due to incompatibility with the FeF_3_ cathode instead. This leaves only the Pyr_13_FSI and TTE/DME electrolyte cells with high capacities at cycle 50, indicating these electrolytes have good compatibility with the FeF_3_ cathode and may be plausible candidates for a full cell electrolyte that can efficiently cycle Li metal and the FeF_3_ cathode.

Further inspection of the data shows that there is a roughly 20% drop in capacity between the first and second discharge (Figure 1 and Figures S1–S5, Supporting Information) for all electrolytes. Similar behavior has been attributed to a combination of 1) irreversible surface reactions which form a cathode electrolyte interphase on the surface of the electrode^[^ ^7^, ^8^, ^10^, ^18^ ^]^ and 2) semireversible changes to the crystal structure of the FeF_3_ upon cycling.^[^ ^19^ ^]^ Evidence for irreversible surface reactions is suggested by the sharp increase in Coulombic efficiency (CE, calculation defined in the Supporting Information) following the first cycle for all cells (Figures S2–S5a, Supporting Information). The CE increases significantly on the second cycle and continues to increase more gradually after that, as observed previously during CEI formation.^[^ ^8^ ^]^ Briefly, the initially low CE indicates a significant number of irreversible reaction are occurring on the initial discharge, likely due to CEI formation. As the passivating CEI layer is further built up, the rate of reaction decreases and the overall cycle reversibility (and thus CE) increases. By cycle 50, the average CE of cells with each electrolyte is 101.5%, 100%, 97.7%, and 99.9% for the Pyr_13_FSI, TTE/DME, bisalt, and EC/DMC electrolytes, respectively, and these near 100% values likely indicate that CEI formation and surface passivation are near completion.

Regarding the semireversible changes to the electrode, Hua et al. have shown that the lithiation of FeF_3_ proceeds through a series of displacement reactions culminating in the conversion of the material to Fe and LiF; recharge proceeds in a mostly reversible manner, but not to completion and therefore not all of the FeF_3_ is regenerated.^[^ ^19^ ^]^ While spectroscopic studies performed by Hua et al. confirm that a similar discharge mechanism involving the same species occurs on discharge for both cycle 1 and 2, a significant change in shape is observed between the two discharge profiles in their work and in ours (Figure S6, Supporting Information). Most notably, the appearance of a clear voltage plateau near 2.25 V on the second cycle likely indicates that while the same species (FeF_2_, A‐Li*_x_* Fe*_y_* F_3_, B‐Li*_x_* Fe*_y_* F_3_) are present in both cycles 1 and 2, their relative abundance, and therefore the overall capacity of the cathode, are different. Furthermore, while the 2.25 V plateau is rapidly lost in the FeF_3_‐incompatible bisalt and EC/DMC cells (Figure S6c,d, Supporting Information), the FeF_3_‐compatible Pyr_13_FSI and TTE/DME cells retain the new plateau over 50 cycles (Figure S6a,b, Supporting Information). Thus, the retention of this new peak appears to be correlated with electrolyte compatibility, likely indicating that the intermediate species formed during cycling are better retained over time in compatible electrolytes. Based on this data, it seems reasonable to classify the mechanism as semireversible, and we infer that some of the capacity loss we observe on cycling our cells may be due to the incomplete reformation of a full FeF_3_ species on recharge and the formation of CEI on the electrode surface.

### 2.2 Electrochemical Li/Electrolyte Compatibility Testing and Rate Cycling

**Figure 2:**
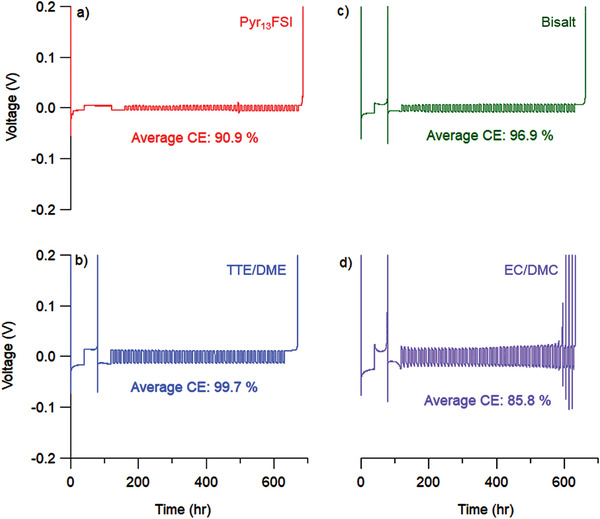
Chronopotentiometric determination of the Coulombic efficiency (CE) of Li plating/stripping in a) Pyr_13_FSI (red), b) TTE/DME (blue), c) bisalt (green), and d) EC/DMC (purple) (*n* = 3 for each). TTE/DME cells show the highest CE, followed by the bisalt and EC/DMC. The Pyr_13_FSI shows variable CE, indicative of poor Li cycling. See the Supporting Information for more details regarding cell construction, experimental procedure, and other considerations.

To verify compatibility with Li, the CE of Li plating and stripping was measured in Li/Cu half‐cells (**Figure** **2** and Figure S7, Supporting Information) using an average CE technique previously reported by Adams et al.^[^ ^20^ ^]^ For this technique, an initial formation cycle was conducted followed by the deposition of a Li reservoir (4 mAh cm^−2^), then a smaller amount of charge (0.5 mAh cm^−2^) was cycled, followed by a final stripping step. By comparing the capacity of the final stripping step to that of the initial Li reservoir, we determined the CE of the Li electrode in each of the four electrolytes [Equation (S2), Supporting Information]. This, in turn, allows us to study the Li compatibility of each electrolyte, independent of the effects of the FeF_3_ cathode. Of the four electrolytes, the TTE/DME and bisalt electrolytes exhibited the highest average CE for Li deposition/stripping (99.7% and 96.9%, respectively), in good agreement with previous results from these electrolytes.^[^ ^14^, ^15^, ^16^ ^]^ In contrast to these high CE values, the EC/DMC cells show a relatively low average CE of 85.8% and exhibits significant polarization toward the end of cycling, indicating poor Li compatibility (Figure 2d). More interestingly, we found that we were unable to reliably determine the CE of Li cycling in the Pyr_13_FSI electrolyte using this technique. The CEs ranged between relatively low values (around 90%) and high values (>100%) during the experiments (Figure S7, Supporting Information). The cell that cycled with an average CE of 90%, exhibited a 200% CE on the formation cycle, suggesting either parasitic reactions or partial shorts early on in cycling. Previous measurements of Li cycling in Li/Li symmetric cells using Pyr_13_FSI suggest good deposition morphology,^[^ ^21^ ^]^ but the cell design precluded the CE measurements needed to accurately judge Li compatibility. Therefore, despite the promising results using Pyr_13_FSI with FeF_2_^8^ and FeF_3_ (Figure 1), the electrolyte appears incompatible with Li and therefore a poor choice for a full cell electrolyte.

This is further confirmed by rate cycling experiments using the Pyr_13_FSI and TTE/DME electrolytes. Despite the two electrolytes showing similar performance at slow cycling rates (C/20–C/5, Figure S8, Supporting Information), the TTE/DME cell out‐performs the Pyr_13_FSI at faster rates (C/2, 1C) and retains its capacity better upon return to slower rates. Additionally, a rate comparison of TTE/DME cells built using either polypropylene or glass fiber separator shows negligible difference in performance (Figure S9, Supporting Information), leading us to conclude that the impact of the glass fiber separator used in the Pyr_13_FSI cells is likely negligible compared to the impact of electrolyte/electrode compatibility. Therefore, while higher rate capability in the TTE/DME relative to Pyr_13_FSI is not unexpected due to its lower viscosity,^[^ ^15^, ^22^ ^]^ we propose that the irreversible capacity loss in the IL electrolyte may be due in part to either the Li‐incompatibility or deleterious effects from the higher viscosity of the IL electrolyte. Regardless of cause, the results clearly show that the rate capability of the TTE/DME electrolyte is superior to that of the Pyr_13_FSI. Taken together, the electrochemical analyses of the FeF_3_‐limited full‐cells, the Li/Cu CE cells, and the rate capability experiments shows that the TTE/DME is the best full cell electrolyte solution among those tested due to its compatibility with both the FeF_3_ cathode and the Li anode. However, as this compatibility could be the result of both physical and/or chemical influences, further characterization is needed to better understand how it differs from the other electrolytes.

### 2.3 SEM Analysis of Electrode/Electrolyte Compatibility

Given the well‐known impact of microstructure on the performance of both anodes and cathodes, we next used SEM to study how the Li and FeF_3_ electrodes were affected by cycling in the various electrolytes. Beginning with the FeF_3_ cathode, secondary electron SEM images collected prior to cycling (Figures S10a and S11a, Supporting Information) show that the cathode film is composed of a mixture of sub‐100 nm carbon particles and sub‐500 nm FeF_3_ agglomerate particles. Backscattered electron images confirm these assignments, as the brighter, high‐Z particles (in this material, Fe‐containing) in Figure S12a (Supporting Information) are a good match to the FeF_3_ agglomerates.

**Figure 3:**
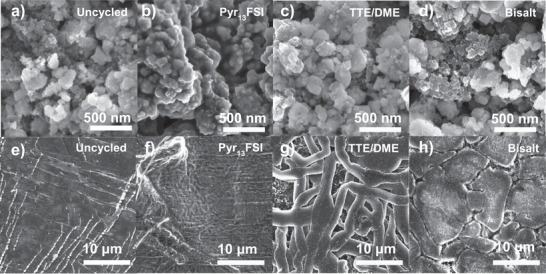
SEM images of an a) uncycled FeF_3_ cathode and identical cathodes cycled in b) Pyr_13_FSI, c) TTE/DME, and d) bisalt electrolyte for two cycles, showing minimal change to microscale morphology. SEM images of an e) uncycled Li anode and identical anodes cycled in f) Pyr_13_FSI, g) TTE/DME, and h) bisalt electrolyte for two cycles, showing the different Li‐deposition morphologies observed in each electrolyte.

After cycling, the overall particle size and morphology of the cathodes do not appear to have been impacted significantly (**Figure** **3**a–d). Similar agglomerate particles are visible before after cycling, and the overall morphology of the TTE/DME, bisalt, and EC/DMC electrodes are largely unchanged (Figures S10 and S11, Supporting Information). In contrast, the Pyr_13_FSI‐cycled cathode shows a thick, conformal layer of material on the surface of the cathode, which appears to be residual IL that was not removed during the wash step;^[^ ^23^ ^]^ backscattered electron SEM images show very little difference in the particles underneath the layer when compared to samples tested in other electrolytes (Figure S12, Supporting Information), supporting this hypothesis. Likewise, further evidence for the composition of this layer can be gleaned from scanning tunneling electron microscopy images of the sample, discussed in a later section. We note, however, that this layer could also be composed, in part or in whole, of CEI products from degradation of the electrolyte on the cathode surface, and that a thicker protective layer of CEI could help explain the capacity retention of FeF_3_ in the Pyr_13_FSI electrolyte. Overall, given the small differences in morphology between the electrodes, but the significant difference in capacity between the carbonate electrolyte cells and the others, it does not appear that the two properties are strongly related.

We next examined the surface morphology of the Li anodes cycled under identical conditions. Prior to cycling, the Li surface appears relatively flat and uniform with some visible lines and nodules, likely consisting of Li_2_O resulting from incidental exposure to atmosphere during the sample loading process prior to SEM analysis (Figures S10c and S13a, Supporting Information). After cycling in each of the electrolytes, we see a variety of different Li deposition morphologies which may be related to the results of the CE measurements above. These differences are visible in both higher‐ (Figure 3) and lower‐magnification images (Figure S13, Supporting Information) of the Li, demonstrating the scale of the differences in Li deposits from each electrolyte. Most notably, Figure 3g,h shows that Li plated from the TTE/DME and bisalt electrolytes consists of relatively large, lower‐surface area deposits, similar to results in previous reports.^[^ ^14^, ^15^ ^]^ In contrast, images of the Li anode from the EC/DMC cell (Figures S10d and S13e, Supporting Information) shows significantly higher surface area, porous deposits that are likely to result in “dead” Li.^[^ ^24^ ^]^ Finally, while pieces of the glass fiber electrode stuck to the Li anode cycled in Pyr_13_FSI, the areas visible through the surface layer of glass fiber separator are largely devoid of clear Li deposits (Figure 3f and Figure S13b, Supporting Information), as described in the Supporting Information, the other cells are constructed using polypropylene separators and do not experience a similar problem upon disassembly. Although Li may have deposited into the glass fiber, these images and the previous CE measurements suggest that parasitic reactions most likely carry the current on Li anodes cycled in Pyr_13_FSI, as opposed to Li plating and stripping; this is in agreement with previous studies showing side reactions and difficulty plating Li in some IL electrolytes.^[^ ^12^, ^21^ ^]^

Overall, these results agree well with the results of the CE measurements in Figure 2 and help bridge the gap between the Li/Cu half‐cell and FeF_3_‐limited full‐cell experiments. The high Li CE measured in the Li‐optimized electrolytes (TTE/DME and bisalt) corresponds to lower surface area deposits when cycled versus FeF_3_, while the low Li CE in the EC/DMC electrolyte corresponds to high surface area Li deposition morphologies. Finally, the lack of Li deposits in the Pyr_13_FSI images strongly suggests that processes other than Li deposition are likely occurring during Li cycling in this electrolyte and that this FeF_3_‐optimized electrolyte may be incompatible with Li, to the detriment of its use as a full cell electrolyte. This is in contrast to what appears to be otherwise excellent cycling performance in our work and in a previous study with of Li/FeF_2_ batteries.^[^ ^8^ ^]^

### 2.4 STEM Analysis of FeF_3_/Electrolyte Compatibility

**Figure 4:**
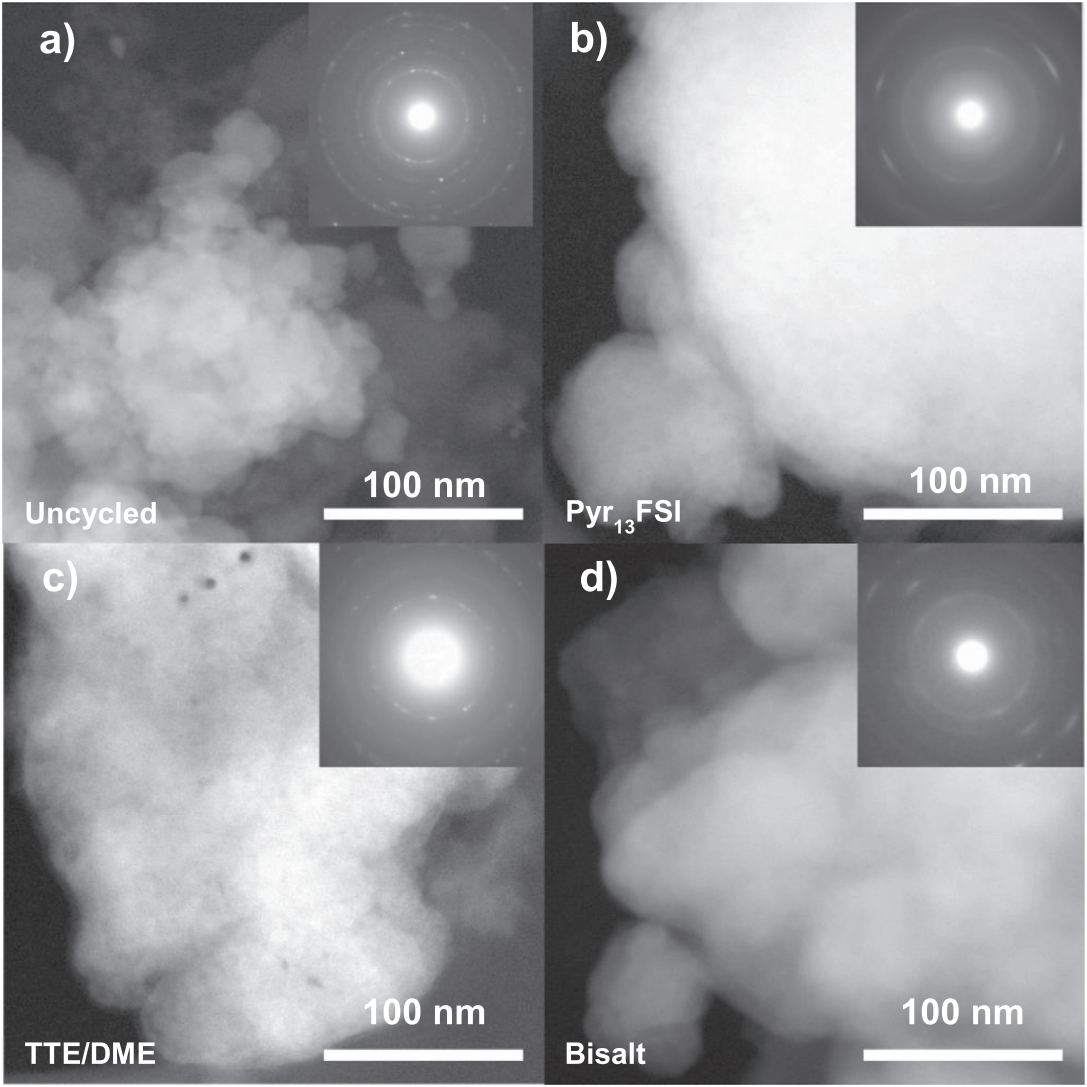
STEM images of a fresh a) FeF_3_ cathode and FeF_3_ cathodes cycled in b) Pyr_13_FSI, c) TTE/DME, and d) bisalt electrolytes for two cycles and stopped on charge, with corresponding SAED patterns (upper right). The primary FeF_3_ particles, roughly 20–50 nm in diameter, are largely lost upon cycling, replaced by smaller (≈5 nm) particles in a less crystalline matrix, indicated by the broadness of the SAED spots and rings in (b)–(d).

While the SEM results discussed above help explain performance differences between the FeF_3_‐ and Li‐optimized electrolytes, they do not differentiate between the TTE/DME and bisalt electrolytes. To better understand the differences between these electrolytes and the others, we used STEM imaging and selected area electron diffraction (SAED) analysis (**Figure** **4** and Figure S14, Supporting Information) of cycled FeF_3_ cathodes to study what role nanostructural changes may play. These higher resolution STEM images show that the agglomerated FeF_3_ particles visible by SEM in uncycled cathodes are composed of highly crystalline (see inset SAED pattern, Figure 4a) particles 20–50 nm in diameter. This is consistent with the 27 nm crystallite size calculated by Scherrer analysis of the X‐ray diffraction spectroscopy (XRD) spectrum of the FeF_3_/C composite after ball milling (Figure S15, Supporting Information), and shows that ball milling enabled the production of small, dispersed FeF_3_ particles, which should improve the electronic and ionic conductivity of the cathode.^[^ ^5^ ^]^

Like in SEM, the STEM images show few changes to the microstructure of the FeF_3_ after two cycles and stopping on charge (Figure S16, Supporting Information), and the agglomerated particles are still clearly visible in the tested samples. For the Pyr_13_FSI sample (Figure S16b, Supporting Information), this is in contrast to the SEM images which showed a relatively thick, conformal layer of material around the FeF*_x_* particles. The absence of this layer in the STEM provides further evidence that the layer is likely composed of residual IL on the surface, which was likely washed away during the comparatively intense sonication used to prepare the sample for STEM analysis, as compared to the gentle washing used to prepare the samples for SEM. The nanoparticles of all samples inside the agglomerates have, however, undergone significant changes; rather than discreet 25 nm crystallites, the secondary microparticles of the cycled cathodes are now composed of smaller, less crystalline 5 nm particles (Figure 4). The spots and rings of the inset SAED patterns are sharpest for the Pyr_13_FSI‐ and TTE/DME‐cycled cathodes, slightly more diffuse for the bisalt cathode, and very diffuse in the EC/DMC‐cycled FeF_3_. This polycrystallinity in the cycled samples is like that observed in previous work,^[^ ^19^, ^25^, ^26^ ^]^ including a recent study from Hua et al. which showed recharged FeF*_x_* cathodes composed of a mixture of nanoscopic, weakly crystalline FeF_3_, FeF_2_, and partially lithiated FeF_3_.^[^ ^19^ ^]^ SAED analysis of our cycled cathodes shows evidence of most of these species in our tested samples (Figure S17, Supporting Information), indicating a similar mechanism is likely at work. Specifically, we observe a transformation from a material predominantly composed of FeF_3_ (containing some FeF_2,_ likely due to ball milling),^[^ ^5^ ^]^ to one largely composed of FeF_2_ with some FeF_3_ and evidence of some metallic Fe, as well.

To better determine the relative composition of the electrodes, we also analyze the Fe L_3_ electron energy loss spectroscopy (EELS) peak of each sample after two cycles (Figure S18, Supporting Information). While the presence of the F K_1_ peak makes a quantitative measurement of Fe^3+^ and Fe^2+^ challenging,^[^ ^25^ ^]^ the peak shape and its position are useful for qualitative assessments. Like the SAED pattern, the EELS spectra of the uncycled FeF_3_ show evidence for both Fe^3+^ and Fe^2+^, with the primary peak near 711 eV indicative of Fe^3+^ and the shoulder at 709 eV corresponding to Fe^2+^.^[^ ^27^ ^]^ In agreement with the higher capacity retention of the Pyr_13_FSI‐cycled FeF_3_, the EELS peak for this twice‐cycled sample shows a primarily Fe^3+^ character that may indicate better FeF_3_ reformation during recharge. The Fe L_3_ EELS peaks of the samples cycled in the TTE/DME and bisalt electrolytes are slightly less predominately Fe^3+^_,_ while the EC/DMC‐cycled cathode shows a primarily Fe^2+^ character. From these data we infer that, as expected based on the battery testing, the Pyr_13_FSI enables the cathode to recharge to FeF_3_ more completely after two cycles than the TTE/DME, bisalt, and EC/DMC electrolytes, and is therefore well‐suited for FeF_3_ cathodes. This advantage is offset, however, by the poor compatibility of Pyr_13_FSI with the Li anode, as observed in Figure 2. These EELS results also indicate that, as the Fe^3+^/Fe^2+^ ratio in the TTE/DME and bisalt electrolyte appears similar, there is likely a chemical, rather than physical, explanation for the improved performance observed with the TTE/DME electrolyte.

### 2.5 XPS Analysis of Electrolyte Impact on CEI and SEI Composition

**Figure 5:**
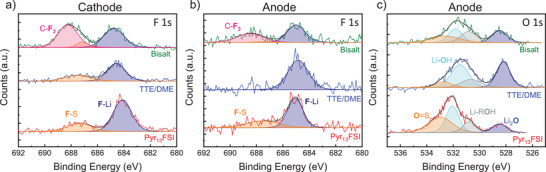
XPS spectra of the a) F 1s region of FeF_3_ cathodes cycled twice in Pyr_13_FSI, TTE/DME, and bisalt electrolytes. The Pyr_13_FSI‐ and TTE/DME‐cycled samples show a more inorganic‐rich CEI than the bisalt. XPS spectra of the b) F 1s and c) O 1s regions of Li anodes cycled twice in the same electrolytes. The TTE/DME‐ and bisalt‐cycled samples show a SEI that is richer in Li_2_O than LiF or LiOH.

Turning next to chemical characterization, we study the surface chemistry of the electrodes after cycling using XPS. Full survey and region spectra of the cathodes and anodes after two cycles are provided in Figures S19 and S20 (Supporting Information), respectively, with the most relevant region spectra shown in **Figure** **5**. Beginning with the F 1s region of the cycled cathodes (Figure 5a), we observe peaks corresponding to LiF (≈684.1 eV),^[^ ^28^ ^]^ fluorosulfonyl (687.4 eV),^[^ ^29^ ^]^ and —CF_3_ species (688.2 eV).^[^ ^28^ ^]^ The lack of Fe 2p peaks in the survey spectra of the cathodes (Figure S19c, Supporting Information) indicates these F species are likely in the surface CEI rather than the bulk of the electrode. However, in the case of the Pyr_13_FSI‐cycled cathode, the XPS provides a final piece of evidence that the CEI layer is likely accompanied by some residual ionic liquid, as previously discussed. Specifically, the pyrrolidinic N peak (401.7 eV) in the N 1s spectra (Figure S19g, Supporting Information), as well as adsorbed S—O species in the O 1s and S 2p spectra (Figure S19e,f, Supporting Information), clearly indicate the presence of Pyr_13_FSI at or near the surface of the cathode. Of the noncarbonate samples, the bisalt CEI is the most organic‐rich due to the large quantity of TFSI‐derived —CF_3_ species, while the Pyr_13_FSI‐ and TTE/DME‐cycled cathodes are predominately composed of the inorganic LiF. LiF is frequently touted for its beneficial properties in Li‐based batteries,^[^ ^30^ ^]^ and thus may provide a Li‐permeable protective barrier to further reaction or Fe dissolution. In contrast, thicker organic‐rich CEI have previously been hypothesized to increase the surface resistance of FeF_3_ cathodes,^[^ ^7^ ^]^ and this may explain why the bisalt cells suffer from poorer performance. We propose then that the TFSI^–^ anion in the bisalt electrolyte breaks down during cycling, producing a CEI rich in insulating —CF_3_ that coats the surface of the FeF_3_ particles. As this layer grows thicker with cycling, it may result in localized regions of high potential capable of inducing damage in the underlying FeF*_x_* cathode. This would ultimately result in the loss of capacity observed in Figure 1 and provide an explanation for the differences in FeF_3_ cycling observed between the Li‐optimized electrolytes.

Next examining the Li anode, the F 1s spectra (Figure 5b) again contain fluorosulfonyl (687.4 eV), —CF_3_ peaks (688.2 eV), and LiF peaks (≈685 eV). Like the CEI, the SEI of the bisalt‐cycled anode is also rich in —CF_3_ species but, unlike the FeF_3_ cathodes, there is little correlation between capacity retention and the relative organic/inorganic composition of the fluorine species in the SEI. Indeed, a comparison of the species present in the SEI of each anode (Figure S20, Supporting Information) shows relatively few compositional differences as a result of cycling in the three electrolytes of interest, with the most notable differing being in the relative concentration of LiF, Li_2_O, and LiOH present in the SEI of the anodes. After cycling, all of the anodes show the presence of Li_2_O (≈528.4 eV),^[^ ^31^ ^]^ Li alkoxides (Li‐ROH, 530.8 eV),^[^ ^32^ ^]^ LiOH (≈531.8 eV),^[^ ^31^ ^]^ and sulfonyl/sulfonimide species (≈532.9 eV)^[^ ^8^ ^]^ in the O 1s spectra (Figure 5c), but the ratio of LiOH to Li_2_O is much lower in the TTE/DME and bisalt anodes compared to the Pyr_13_FSI anodes (1.1:1 and 2.8:1, respectively). Additionally, the TTE/DME and bisalt anodes also show greater quantities of Li_2_O in the SEI relative to LiF; the Li_2_O/LiF ratio for the Li‐optimized electrolytes is roughly 1.3:1, compared to 0.63:1 for Pyr_13_FSI.

While there is still some question as to whether LiF is beneficial or detrimental to the electrochemical cycling of Li metal anodes,^[^ ^30^ ^]^ prior work has shown that Li_2_O tends to have higher Li conductivity than LiF or LiOH^[^ ^33^, ^34^ ^]^ and that Li vacancies at the interface of an Li_2_O/Li system are easier to fill than those in a LiF/Li system.^[^ ^35^ ^]^ Given the poor cycling behavior of the Li in Pyr_13_FSI, it is possible that the poor properties of the SEI formed in this electrolyte may contribute to increased surface potential, decreased Li deposition, and parasitic side reactions that produce soluble products which may diffuse away from the anode surface. We note that Li stripping and plating remains a complicated topic of research, dependent on both physical and chemical factors.^[^ ^36^ ^]^ Therefore, additional research is needed to better understand of how specific IL electrolytes interact with Li metal anodes. At present, however, our analysis of the Li metal anodes using electrochemistry, SEM, and XPS indicates that the proper ratio of inorganic species in the SEI is important to ensure the good stability, and therefore CE, of Li metal anodes in full‐cells versus FeF_3_ cathodes.

### 2.6 Electrolyte Impact on Cycling of Limited‐Li Full Cells

**Figure 6:**
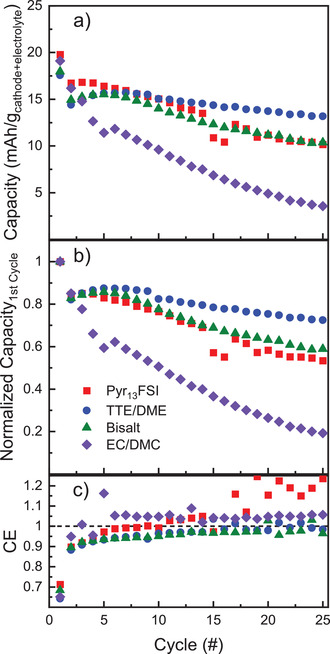
a) Average discharge capacity of FeF_3_/Li cells, tested using limited‐Li metal anodes (20 µm) in Pyr_13_FSI, TTE/DME, bisalt, and EC/DMC electrolytes at C/20, reported per gram of cathode and electrolyte. b) Normalized average discharge capacity of cells in (a), demonstrating the increased stability of the TTE/DME cells. c) Average CE of cells reported in (a), demonstrating the poor cycling stability of the Pyr_13_FSI and EC/DMC cells. Average values and standard deviation are calculated from three cells (*n* = 3), and error bars are omitted here for clarity.

To further validate our conclusions above, we conducted galvanostatic testing of Li‐limited full‐cells in each of the electrolytes (**Figure** **6**). These cells are identical to those tested in Figure 1, but are built using a 20 µm thick Li anode rather than 750 µm to better balance the anode and cathode capacities (≈3‐fold excess Li capacity, Table S1, Supporting Information). Given that the cells are still cathode‐limited and that the choice of electrolyte is integral to cell performance, we have presented the cell capacity per grams of both cathode and electrolyte (Figure 6a). Doing so allows us to better capture the impact of electrolyte choice on the performance of these full cells. Additionally, we have presented the discharge capacity normalized to the first cycle capacity in Figure 6b, to better highlight the differences in capacity retention between the electrolytes. The corresponding charge capacity plots are provided in Figure S21 (Supporting Information), while the average discharge capacity per gram of FeF_3_ alone is provided in Figure S22 (Supporting Information).

Based on our previous experiments, one would expect the EC/DMC cells to rapidly fail as both the Li anode and FeF_3_ cathode experience unfavorable cycling conditions, while the Pyr_13_FSI and bisalt cells should retain capacity significantly longer due to the failure of only a single electrode; the TTE/DME cell is expected to show the slowest rate of decay, due to the compatibility of the electrolyte with both electrodes. As expected, although all of the cells show similar initial discharge capacities (Figure 6a), the EC/DMC cell drops below 50% of the initial charge/discharge capacity within ten cycles. while the bisalt cell shows a slow decay similar to that observed when using excess Li (Figure 6b). Although the discharge capacity of the Pyr_13_FSI cell shows what appears to be a gradual, steady decline with cycle number, there is a sharp increase in the CE of the cell above 100% beginning after cycle 10 that is accompanied by increasingly erratic CE measurements (Figure 6c). This behavior is likely due to the parasitic side reactions on the Li anode previously discussed, and indicates that the thin Li anode is being damaged or poorly‐cycled in this electrolyte. Only the TTE/DME cell shows both slow capacity loss and uniform charge/discharge behavior indicative of the best compatibility with both electrodes. Thus, our data shows that Li/FeF_3_ batteries are well optimized in TTE/DME electrolytes due to efficient cycling of both electrodes. Taken all together, these results presented in this work support the need for careful consideration of the compatibility of all components of a full‐cell battery when testing new materials in a half‐cell format. These data also show that Li/FeF_3_ batteries are well optimized in the TTE/DME electrolyte, enabled by efficient cycling of both electrodes.

## 3 Conclusions

Using electrochemical, STEM, XPS, and other characterization techniques, we have shown that a locally concentrated electrolyte solution composed of LiFSI dissolved in a mixed TTE/DME solvent is a promising full cell solution for Li metal anode/FeF_3_ cathode batteries. The electrolyte enables efficient, stable cycling of the FeF_3_ cathode over 50 cycles, while simultaneously cycling Li metal with a high (>99%) CE. Other electrolytes tested, including solutions based on Pyr_13_FSI, concentrated bisalt, and a typical carbonate electrolyte, showed good performance for only the cathode or the anode alone, precluding their use in stable full‐cell systems pairing Li metal anodes with FeF_3_ conversion cathodes. SEM analysis confirmed minimal microscopic structural changes to the FeF_3_ cathode, and micrometer‐scale low surface area Li deposits on the anode for electrodes cycled in the TTE/DME. Subsequent STEM images showed irreversible changes to the FeF_3_ material after cycling in all electrolytes, resulting in an active mixture containing both Fe^3+^ and Fe^2+^ species after the first cycle. XPS analysis of the cycled anodes and cathodes confirmed that, upon cycling, the TTE/DME electrolyte produced an inorganic‐rich CEI on the cathode and a Li_2_O‐rich SEI on the anode that likely allowed for the stable cycling of both materials in the same electrolyte; the other electrolytes tested were only capable of producing an appropriate protective surface on one electrode, limiting their efficacy as full cell electrolytes. This result demonstrates the importance of taking the electrolyte compatibility of both the Li metal anode and the conversion cathode into account when studying new electrolytes or conversion materials for full‐cells, rather than simply studying FeF*_x_* or Li half‐cell architectures alone. Future work on FeF_3_ and the many other conversion cathode candidates should make note of these considerations to more accurately determine the promise of new full‐cell systems.

## 4 Experimental Section

### Electrolyte and Cell Preparation

Full details about the materials used, the preparation of the FeF_3_/C composites and cathodes, and the preparation of the various electrolytes are available in the Supporting Information. Briefly, a FeF_3_/C composite was prepared by ball milling and used to create cathodes with a 70:20:10 ratio of FeF_3_/carbon/binder on C‐coated Al foil. Capacities were reported per gram of active material (FeF_3_) in the initial electrode, unless otherwise indicated. The following electrolyte solutions were prepared in an Ar‐filled glovebox (MBraun) with O_2_/H_2_O levels < 0.2 ppm: 1 m LiFSI in Pyr_13_FSI, 1.73 m LiFSI in a 3.65:1 (v/v) solution of DME/TTE, 2 m LiFSI, and 1 m LiTFSI in a 1:1 (v/v) solution of DOL/DME, and 1 m LiPF_6_ in a 1:1 (v/v) solution of EC/DMC. 2032 coin cells were constructed in the same glovebox as per Figure S23 (Supporting Information), with the materials detailed in Table S1 (Supporting Information). Cells for testing the CE were constructed using a 50 µm‐thick Li electrode on Cu versus a 9 µm‐thick Cu electrode, while all other cells consisted of an FeF_3_ cathode paired with either a 750 µm‐ or 20 µm‐thick Li anode. The same quantity of electrolyte was used in both 750 µm and 20 µm Li cells. With the exception of rate cycling tests, all cells were tested in triplicate.

### Material Characterization

Full details about the instruments and settings used for characterization are available in the Supporting Information. In summary, galvanostatic testing was performed to evaluate the performance of the various electrochemical cells tested using each of the four electrolytes. All FeF_3_/Li cells were cycled at a rate of C/20 relative to the total capacity of the FeF_3_ cathode, unless otherwise specified. Li CE tests were conducted according to “Method 3” as previously reported.^[^ ^20^ ^]^ For detailed analysis of the electrodes after testing in FeF_3_/Li cells, the Li anodes and FeF_3_ cathodes were harvested from cells after two cycles, and rinsed with dry DME to remove any adsorbed electrolyte. The Li anodes were analyzed using SEM and XPS, while the FeF_3_ cathodes were analyzed using SEM, STEM, EELS, XRD, and XPS. Details on statistical analysis of the results are provided in the Supporting Information.

## Conflict of Interest

The authors declare no conflict of interest.

## Supporting information

Supporting Information

## Data Availability

Research data are not shared.
