# Supplementary material for: The Role of Electrolyte Composition in Enabling Li Metal‐Iron Fluoride Full‐Cell Batteries
Source: Adv Sci (Weinh). 2022 Feb 24;9(12):2105803. doi: 10.1002/advs.202105803 (PMC9036002; doi:10.1002/advs.202105803)
Supplement: Supplementary file 1 — Supporting Information [file ADVS-9-2105803-s001.pdf]

ADVANCED SCIENCE
Open Access

Supporting Information

for *Adv. Sci.*, DOI 10.1002/advs.202105803

The Role of Electrolyte Composition in Enabling Li Metal-Iron Fluoride Full-Cell Batteries

*Bryan R. Wygant, Laura C. Merrill, Katharine L. Harrison, A. Alec Talin, David S. Ashby and Timothy N. Lambert**

Supplemental Information for:

The Role of Electrolyte Composition in Enabling Li Metal-Iron Fluoride Full-Cell Batteries

Bryan R. Wygant,[a] Laura C. Merrill,[b] Katharine L. Harrison,[a] A. Alec Talin,[c] David S. Ashby,[c] Timothy N. Lambert[a]

[a]Department of Photovoltaics and Materials Technology, Sandia National Laboratories, Albuquerque, New Mexico, 87185, United States

[b]Department of Nanoscale Sciences, Sandia National Laboratories, Albuquerque, New Mexico, 87185, United States

[c]Department of Quantum & Electronic Materials, Sandia National Laboratories, Livermore, California, 94550

*tnlambe@sandia.gov

Detailed Experimental Conditions

Materials and Electrolyte Preparation

Unless otherwise specified, all materials were used as received. Anhydrous FeF_3 powder (Alfa Aesar, 97%) and carbon black (Super P) were added to a 45-mL stainless steel ball mill cup (Fritsch) in a 3.508:1 mass ratio inside an Ar-filled glovebox (MBraun) with H_2O/O_2 less than 5 ppm. A 20:1 mass ratio of 3 mm stainless steel milling media (relative to the total mass of FeF_3 and carbon) was then added, and the cup was sealed under an Ar atmosphere. The material was then milled at 1000 rpm for 30 min using a Fritsch Pulvrisette 7 ball mill before being unloaded from the cup and stored inside the glovebox.

All electrolytes were prepared inside an Ar-filled glovebox (MBraun) with H_2O/O_2 concentrations less than 2 ppm. Prior to preparation, anhydrous 1,2-dimethoxyethane (DME, Sigma Aldrich), anhydrous 1,3-dioxoloane (DOL, Sigma Aldrich), and 1,1,2,2-tetrafluoroethyl 2,2,3,3-tetrafluoropropyl ether (TTE, Synquest Laboratories) were dried over activated alumina for at least 48 hours. Lithium bis(fluorosulfonyl)imide (LiFSI, Oakwood Chemical) and lithium bis(trifluoromethanesulfonyl)imide (LiTFSI, Ossila) were dried at 100 °C under vacuum in a heated glovebox antechamber for at least 48 h prior to electrolyte preparation. To prepare the $Pyr_{13}FSI$ electrolyte, 5 mmol of LiFSI was dissolved in 5 mL of n-propyl-n-methylpyrrolidinium bis(fluorosulfonyl)imide ($Pyr_{13}FSI$, Solvionic 99.9%) by stirring overnight in an Ar-filled glovebox. To prepare the DME/TTE electrolyte, 2.94 mmol of LiFSI was dissolved in 318 mg of DME and 2.05 g of TTE by stirring overnight to form a 1 LiFSI:1.2 DME:3TTE molar ratio.[1] The Bisalt, 2 M LiFSI:1 M LiTFSI in a 50/50 (v/v) mixture of DOL/DME, was prepared in a volumetric flask to account for any volumetric changes associated with salt dissolution. A

solution of 1M $LiPF_6$ in a 1:1 (v/v) ratio of ethylene carbonate/dimethyl carbonate (EC/DMC) from Sigma Aldrich was used as received as the EC/DMC electrolyte.

Cathode Preparation

All cathode preparation was performed inside a glovebox under an Ar atmosphere. A slurry containing a 70:20:10 ratio of FeF_3/carbon/binder in 1-methyl-2-pyrrolidinone (NMP, Sigma Aldrich, anhydrous) was prepared by stirring over 2 hours. Polyvinylidene fluoride (PVDF, 5130 Solvay) was first dissolved completely in the NMP before dispersing the FeF_3/C. Typical slurries had a solid content of 200 mg/mL of NMP. After mixing, the slurry was coated onto a piece of carbon-coated Al foil by doctor blading using a 20 µm slot. The film was dried at 65 °C overnight, then 1.98 cm^2 electrodes were punched from the substrate and used as cathodes in the batteries. The electrodes had an average areal loading of 2.44 mg_{FeF3}/cm^2 and an average areal capacity of 1.74 mAh/cm^2.

Full-Cell FeF_3/C Battery Assembly

Please see **Figure S20** for a detailed graphic of the coin cells described below and **Table S1** for physical properties of the various cell components. All coin cells were constructed inside an Ar-filled glovebox (MBraun) with H_2O/O_2 concentrations less than 0.2 ppm. For all electrolytes, CR2032 coin cells were constructed using a 1.98 cm^2 FeF_3/C cathode and a 2.01 cm^2 Li anode; excess-Li cells used a 750 µm-thick anode (Alfa Aesar) and limited-Li cells used a 20 µm-thick anode on 10 µm Cu foil (Albermarle). In addition, a single wave spring and 0.7 mm of stainless steel spacer were also included inside the cell casing for 750 µm Li cells, while a single wave spring and 1.2 mm of stainless steel spacer were used for 20 µm Li cells. The same volume of electrolyte and number/type of spacer were used for both 750 µm and 20 µm Li cells. For cells containing $Pyr_{13}FSI$ electrolyte, 100 µL of electrolyte and a single 2.84 cm^2 glass fiber separator

(Whatman, GF/C) was used. For cells containing the TTE/DME and bisalt electrolytes, 70 μL of electrolyte and two 2.84 cm^2 polypropylene separator (Celgard 2400) were used. For the EC/DMC electrolyte, 70 μL electrolyte and two 2.84 cm^2 polymer separators (W-SCOPE) were used. Separators were chosen based on their wettability by each electrolyte; EC/DMC does not wet Celgard 2400 as well as W-SCOPE, while $Pyr_{13}FSI$ does not wet either polypropylene separator, instead requiring the use of glass fiber to allow for adequate wetting.

Half-cell Li/Cu Construction

Lithium-copper half-cells were prepared for Li plating Coulombic efficiency measurements. Unless otherwise specified, all materials were used as received. Cu working electrodes (9 μm) were treated with a 1.2 M HCl bath to remove the native oxide. Following the HCl bath the Cu electrodes were washed with 18 MΩ water and then acetone. The Li counter electrodes, unless otherwise noted, were a 50 μm Li metal laminate on 10 μm of Cu foil (Albermarle). 750 μm Li (Alfa Aesar), used in specified tests, was also used. Electrodes were punched into 16 mm discs and had an area of 2.01 cm^2. The coin cells prepared with the TTE/DME and bisalt electrolytes used two Celgard 2400 separators with 80 μL of electrolyte and the coin cells prepared with EC/DMC used two W-SCOPE separators with 80 μL of electrolyte; these cells all used the 50 μm-thick Li. The coin cells prepared with the $Pyr_{13}FSI$ electrolyte used one glass fiber separator (Whatman, GF/C) and 100 μL of electrolyte and the 750 μm-thick Li. As previously mentioned, the choice for each separator was based on the wettability of each separator material with each electrolyte. The use of the 750 μm-thick Li with the ionic liquid electrolyte was based on supplemental lithium cycling measurements as discussed below.

Physical Characterization

Powder X-ray diffraction (XRD) spectroscopy was performed on a D2 Phaser (Bruker) spectrometer using a Cu kα radiation source. Cells used for physical characterization were cycled twice and stopped at a charged state (4.0V) before being opened inside the Ar-filled glovebox to harvest cathodes and anodes. The electrodes were soaked in DME for 5 seconds to remove any residual electrolyte from the surface and allowed to dry prior to further processing. Scanning electron microscopy (SEM) images were collected using a Supra 55VP Field Effect Scanning Electron Microscopy (Zeiss) at 3 kV accelerating voltage using both an electron backscatter detector (EBSD) and a secondary electron detector. All samples were loaded into the microscope using an Ar-filled glovebag to limit exposure of the samples to oxygen and moisture. X-ray photoelectron spectroscopy (XPS) was performed using a K-alpha X-ray photoelectron spectrometer (Thermo Scientific) using a monochromatic Al source (Kα = 1486.6 eV). Samples were loaded into the XPS using an inert atmosphere transfer arm to prevent exposure to moisture and oxygen. All spectra were corrected to an adventitious (sp3) carbon peak at 284.8 eV. Scanning transmission electron microscopy (STEM) images were captured using a Titan G2 80-200 microscope (FEI Company) operated at 200 kV and equipped with four silicon-drift X-ray detectors (Super X^{TM}). Electron energy loss spectra (EELS) were collected using a Quantum 963 spectrometer (Gatan, Inc.). Samples for STEM analysis were prepared by sonicating a small piece of the cathode film in dry ethanol inside a sealed bottle to suspend some of the FeF_3/C composite, then cast onto lacey carbon grid and allowed to dry inside the Ar glovebox. They were then transferred into the STEM instrument inside an inert atmosphere transfer arm to limit exposure to ambient atmosphere and moisture.

Electrochemical Characterization

Full-cell FeF_3/Li cells and limited Li cells were tested galvanostatically on a Series 4000 battery tester (Maccor). Except where noted, cells were cycled between 1 and 4 V at a C/20 rate relative to the capacity of the FeF_3 in the cathode. Average cell capacities were calculated from triplicate measurements of identically constructed and cycled cells using each electrolyte, and were reported with error bars equivalent to a single standard deviation from the mean. For FeF_3/Li cells, Coulombic efficiency is defined according to **Equation S1**.

Equation S1:

$$CE = \frac{Charge\ Capacity_{Cycle\ "n"}\ (\frac{mAh}{g})}{Disharge\ Capacity_{Cycle\ "n"}\ \left(\frac{mAh}{g}\right)}\ x\ 100\%$$

The lithium Coulombic efficiency measurements were made using "Method 3" as described by Adams, *et al.* in their previous work using Arbin battery cyclers.[2] First, a lithium reservoir of 4 mAh/cm^2 was plated at 0.1 mA/cm^2, next the reservoir was stripped, and replated. Then 0.5 mAh/cm^2 of charge was cycled 51 times before the final, exhaustive stripping step. A +/- 1 V voltage limit was applied for the stripping/plating steps. The average Coulombic efficiency was calculated according to **Equation S2**,as described in the literature[3]:

Equation S2:

$$CE_{avg} = \frac{nQ_c + Q_s}{nQ_c + Q_T}$$

where n is the number of cycles, Q_T is the amount of charge plated in the lithium reservoir, Q_c was the amount of charge passed through over the 51 cycles, and Q_s was the final amount of charge stripped. For cells that polarized (and therefore could not deliver the designated amount of charge) before reaching the 51 cycles, the total amount of plating and stripping charge were summed over

the 51 cycles and that value replaced the nQ_c term in the equation. The charge from the first, or formation cycle, is not included in this calculation.

Some supplementary lithium/copper cycling tests were also completed with the $Pyr_{13}FSI$ electrolyte. For these tests, 0.5 mAh/cm^2 of lithium was plated at 0.1 mA/cm^2 (or until it reached a -1 V voltage cutoff) and then lithium was stripped until it reached a 1 V limit. A maximum time limit, resulting in twice the amount of charge (1 mAh/cm^2), was used on the stripping step to identify soft short circuits or parasitic reactions that artificially inflate the Coulombic efficiency measurements.

Cells used for SEM, STEM, and XPS analysis were discharged and charged twice at C/20 prior to being opened inside an Ar glovebox. The anode and cathode were harvested from the opened cells and dunked in DME for 5 seconds to remove any residual electrolyte solution and left to dry.

Statistical Analysis

1) Pre-processing of data: Capacities measured from cycling data was transformed from units of "mAh" reported by the instrument to "mAh/g" by dividing this capacity by the mass of the FeF_3 in the cell's cathode, for the excess-Li cells, or by the total mass of the cathode and the electrolyte together, for the limited-Li cells. For XPS measurements, a Shirley background was used to fit all of the spectra and subsequently subtracted before plotting. 2) Data presentation: All graphs presenting the averaged results of electrochemical tests (e.g. **Figure S1**) are presented as the mean +/- standard deviation (SD), with error bars or shaded areas representing the standard deviation. 3) Sample size: All electrochemical tests were performed in triplicate (n = 3); all physical characterizations were performed on a single sample (n = 1). 4) Statistical methods: No

statistical differentiation was performed in this work. 5) Software: Data was processed in Microsoft Excel, Origin Labs Origin 2020, and KasaXPS.

Supplementary Discussion: Coulombic Efficiency Measurements using $Pyr_{13}FSI$

Supplemental Li plating and stripping measurements using $Pyr_{13}FSI$ electrolyte were also conducted (**Supplemental Discussion Figure 1**). For these measurements, Li was plated and stripped on a Cu working electrode at 0.1 mA/cm^2 for 0.5 mAh/cm^2. Both thin (50 µm) and thick (750 µm) Li were cycled under these parameters to determine the Coulombic efficiency of Li cycling. We found that the cell using the 750 µm Li is able to cycle reasonably for the first 10 cycles but the cells with 50 µm Li had Coulombic efficiencies greater than 100 % as early as the first cycle. This suggests that Li is not able to effectively cycle using the thinner 50 µm Li with the $Pyr_{13}FSI$ electrolyte, and therefore the 750 µm Li was used for the average Coulombic efficiency measurements. Even still, the average Coulombic efficiency measurements of the $Pyr_{13}FSI$ electrolyte resulted in significant variability and unreliable measurements, exemplified by greater than 100 % CE on the formation cycle with 90 % average CE, as well as an average calculated CE that is greater than 100 % efficiency (see **Figure S7** for replicates). Furthermore, the overpotential was much lower than that of the other electrolytes (around 3 mV versus around 30 mV overpotentials for lithium plating). While this low overpotential could indicate that plating and stripping is less kinetically limited in the $Pyr_{13}FSI$ electrolyte, it more likely suggests soft shorts, given the other data that points to difficulty cycling. The cause of the sporadic behavior with the 750 µm Li may be due to parasitic reactions during cycling or Li growth

through the glass fiber separator leading to partial shorts within the cell. Ultimately it was determined that the $Pyr_{13}FSI$ electrolyte can cycle Li metal with moderately low efficiencies (80-91 %) for a limited number of cycles but failure is impending.

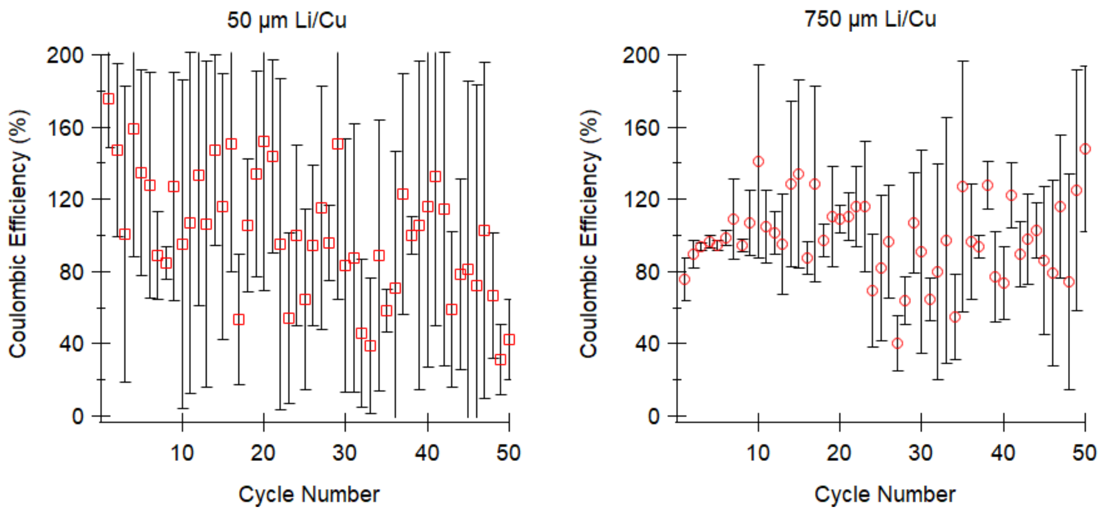

Supplemental Discussion Figure 1: Coulombic efficiency vs cycle number of Li plating and stripping on Cu working electrode in $Pyr_{13}FSI$ electrolyte. Li was cycled at 0.1 mA/cm^2 for 0.5 mAh/cm^2. Left – 50 µm Li and Right – 750 µm Li. Averages shown here are of 3 cells (n = 3).

Table S1: Physical properties of components used in construction of CR2032 coin cells for full cell testing

Component	Mass (g)	Thickness (mm)	Diameter (mm)	Capacity (mAh)
FeF_3 Cathode (+ Al current collector)	0.0140	0.066	16.0	3.437
750 μm Li Anode	0.0805	0.75	16.0	310.880
20 μm Li Anode	0.0042	0.03	16.0	8.293
Glass Fiber Separator	0.0147	0.19	19.0	X
Celgard Separator	0.0041	0.029	19.0	X
W-scope Separator	0.0023	0.015	19.0	X
0.1 mm spacer	0.2913	0.1	15.5	X
0.5 mm spacer	0.8173	0.5	16.0	X
Cathode Cap	0.908	X	X	X
Anode Cap	0.8927	X	X	X
Wavespring	0.1673	X	X	X

Table S2: Gravimetric and Volumetric capacities of CR2032 full-cells tested. Mass and volume are based on all non-case components (lids, springs, and spacers) of the cell, including the anode, cathode, separator(s), and electrolyte; current collectors and inactive electrode constituents are included.

Electrolyte	Gravimetric Energy Density, 750/20 μm Li (mWh/kg)	Volumetric Energy Density, 750/20 μm Li (mWh/L)
$Pyr_{13}FSI$	117/165	97/177
TTE/DME	138/210	123/291
Bisalt	142/218	123/291
EC/DMC	159/262	127/314

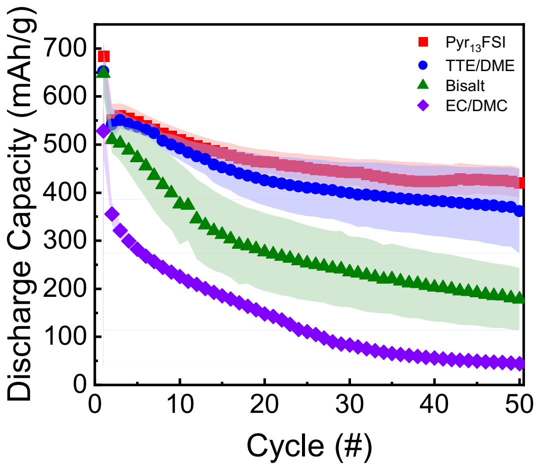

Figure S1: Average discharge capacity of FeF_3/C cathodes versus 750 μm Li metal at C/20 in $Pyr_{13}FSI$, TTE/DME, bisalt, and EC/DMC electrolytes. Cells cycled in $Pyr_{13}FSI$ and TTE/DME show comparable performance, while both the bisalt and EC/DMC show significantly worse capacity retention over 50 cycles. Average values and standard deviation are calculated from 3 cells (n = 3), and presented as mean±SD.

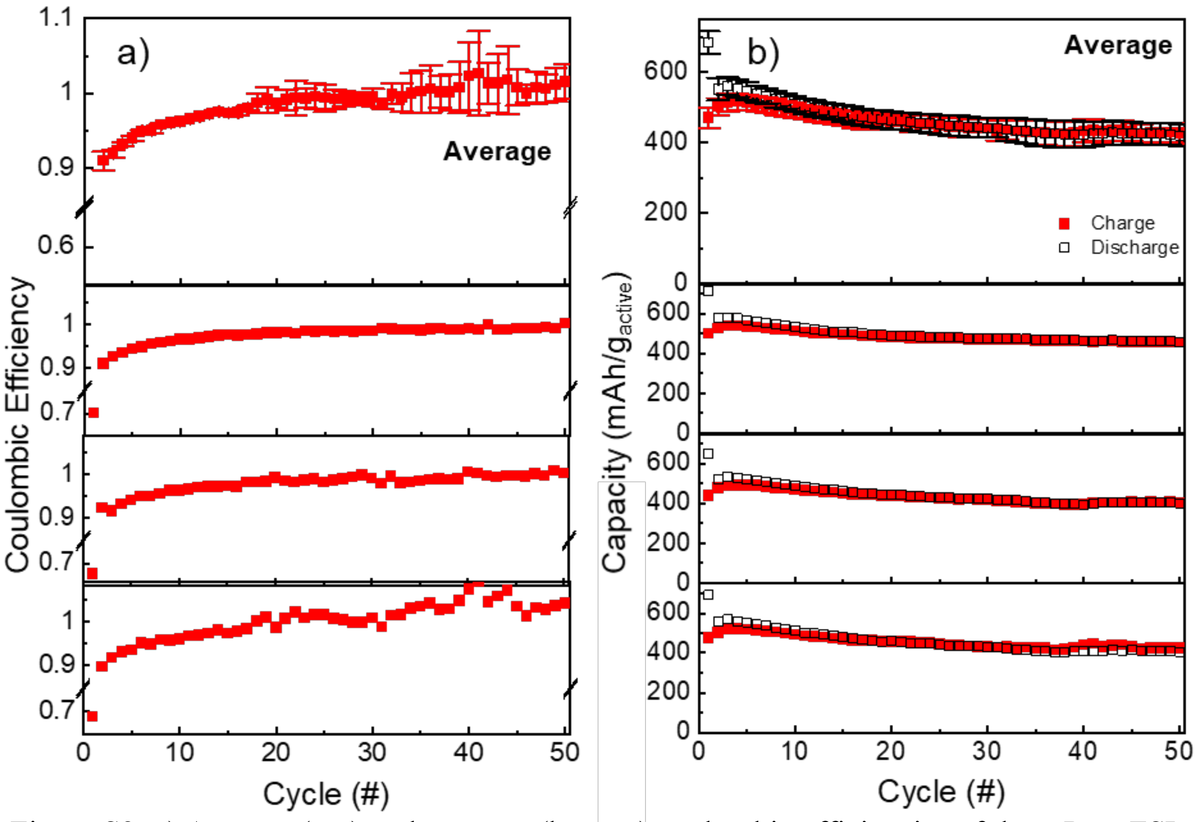

Figure S2: a) Average (top) and separate (bottom) coulombic efficiencies of three $Pyr_{13}FSI$ coin cells cycled at C/20. b) Average (top) and separate (bottom) charge and discharge capacities of three $Pyr_{13}FSI$ coin cells. Average values and standard deviation are calculated from 3 cells (n = 3), and presented as mean±SD.

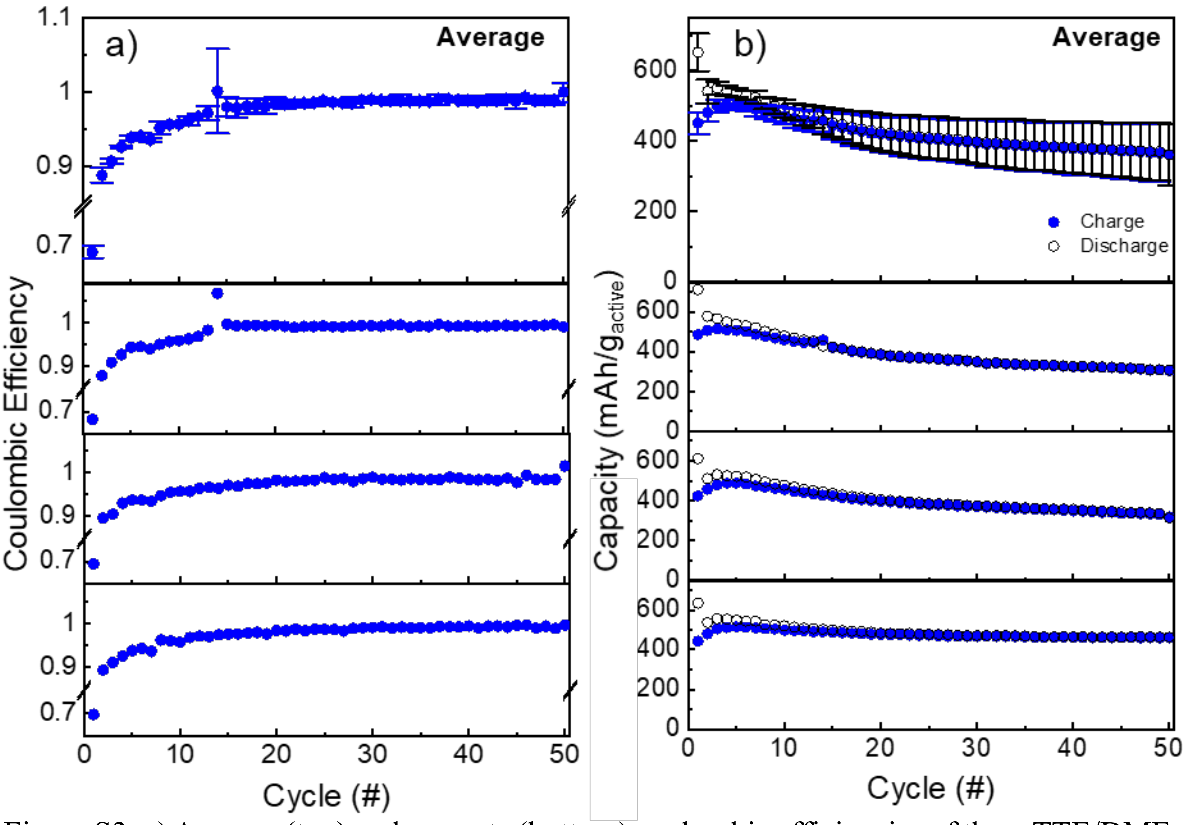

Figure S3: a) Average (top) and separate (bottom) coulombic efficiencies of three TTE/DME coin cells cycled at C/20. b) Average (top) and separate (bottom) charge and discharge capacities of three TTE/DME coin cells. Average values and standard deviation are calculated from 3 cells (n = 3), and presented as mean±SD.

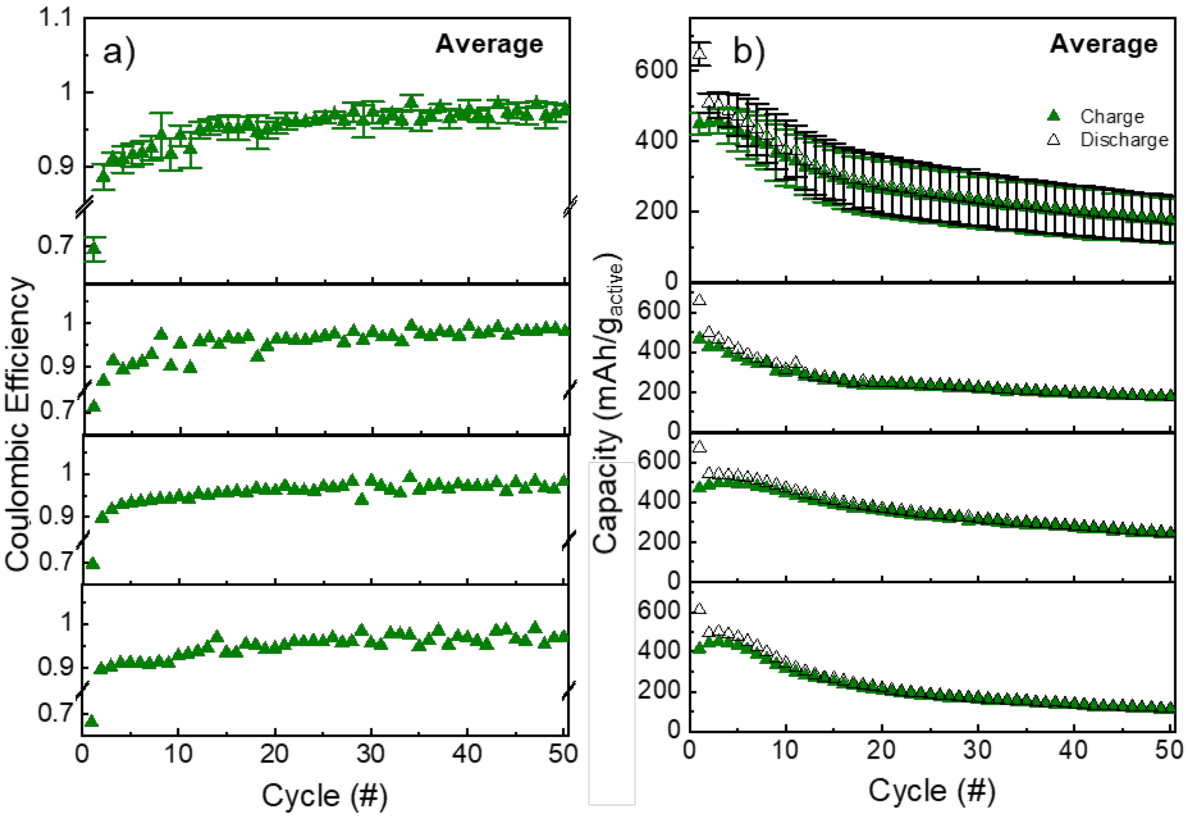

Figure S4: a) Average (top) and separate (bottom) coulombic efficiencies of three bisalt coin cells cycled at C/20. b) Average (top) and separate (bottom) charge and discharge capacities of three bisalt coin cells. Average values and standard deviation are calculated from 3 cells (n = 3), and presented as mean±SD.

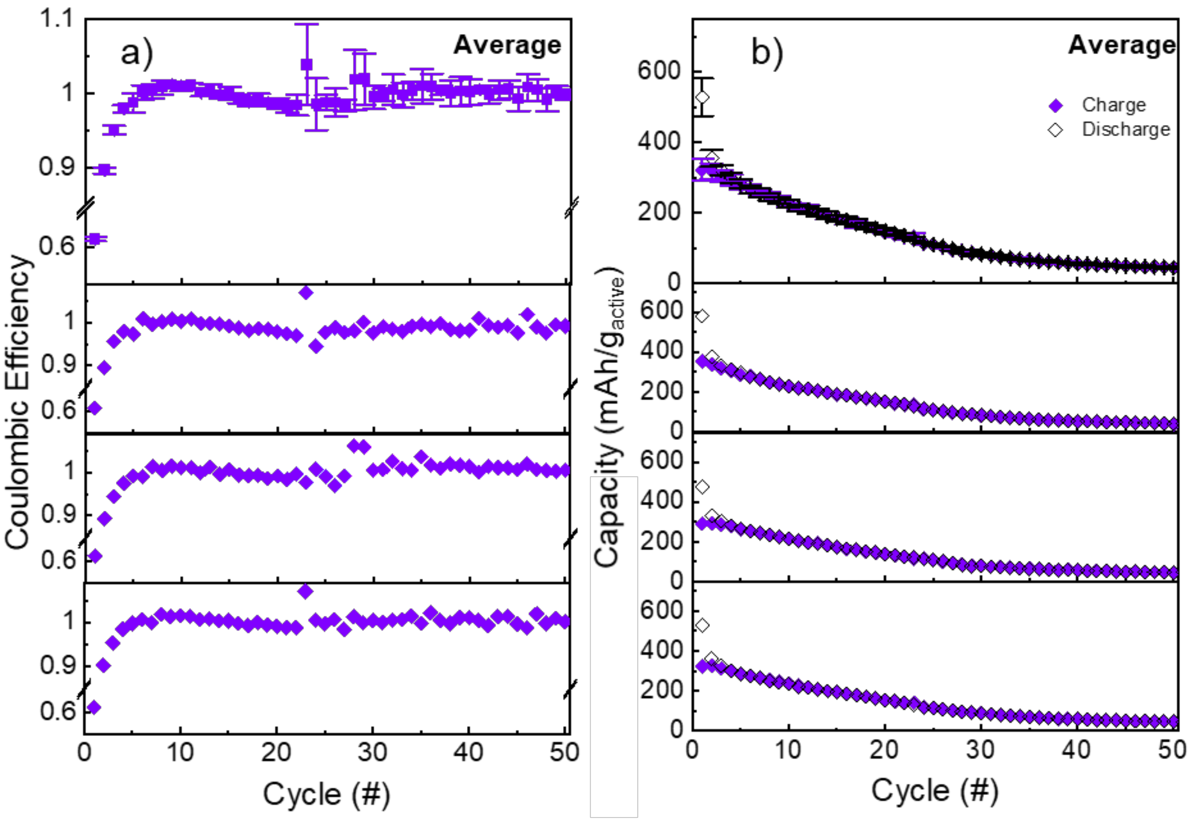

Figure S5: a) Average (top) and separate (bottom) coulombic efficiencies of three EC/DMC coin cells cycled at C/20. b) Average (top) and separate (bottom) charge and discharge capacities of three EC/DMC coin cells. Average values and standard deviation are calculated from 3 cells (n = 3), and presented as mean±SD.

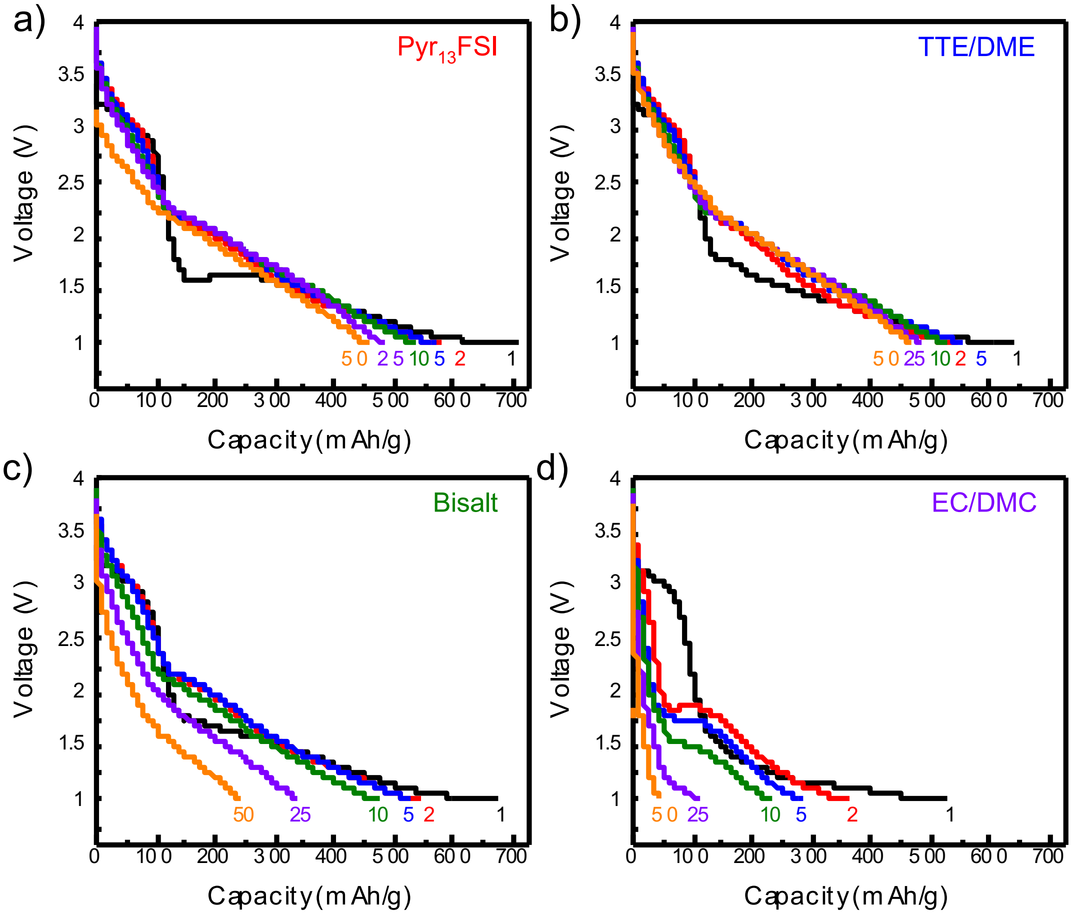

Figure S6: Charge/discharge plots for the 1st, 2nd, 5th, 10th, and 50th cycles of a) $Pyr_{13}FSI$, b) TTE/DME, c) bisalt, and d) EC/DMC coin cells cycled at C/20. All cells show a distinct two-phase initial discharge with plateaus at approximately 3.1 V and 1.6 V; this second plateau moves to 2.25 V in subsequent discharges. The initial charge shows two plateaus and appears similar to subsequent charge steps, though a third plateau gradually appears around 2.25 V after cycle 10.

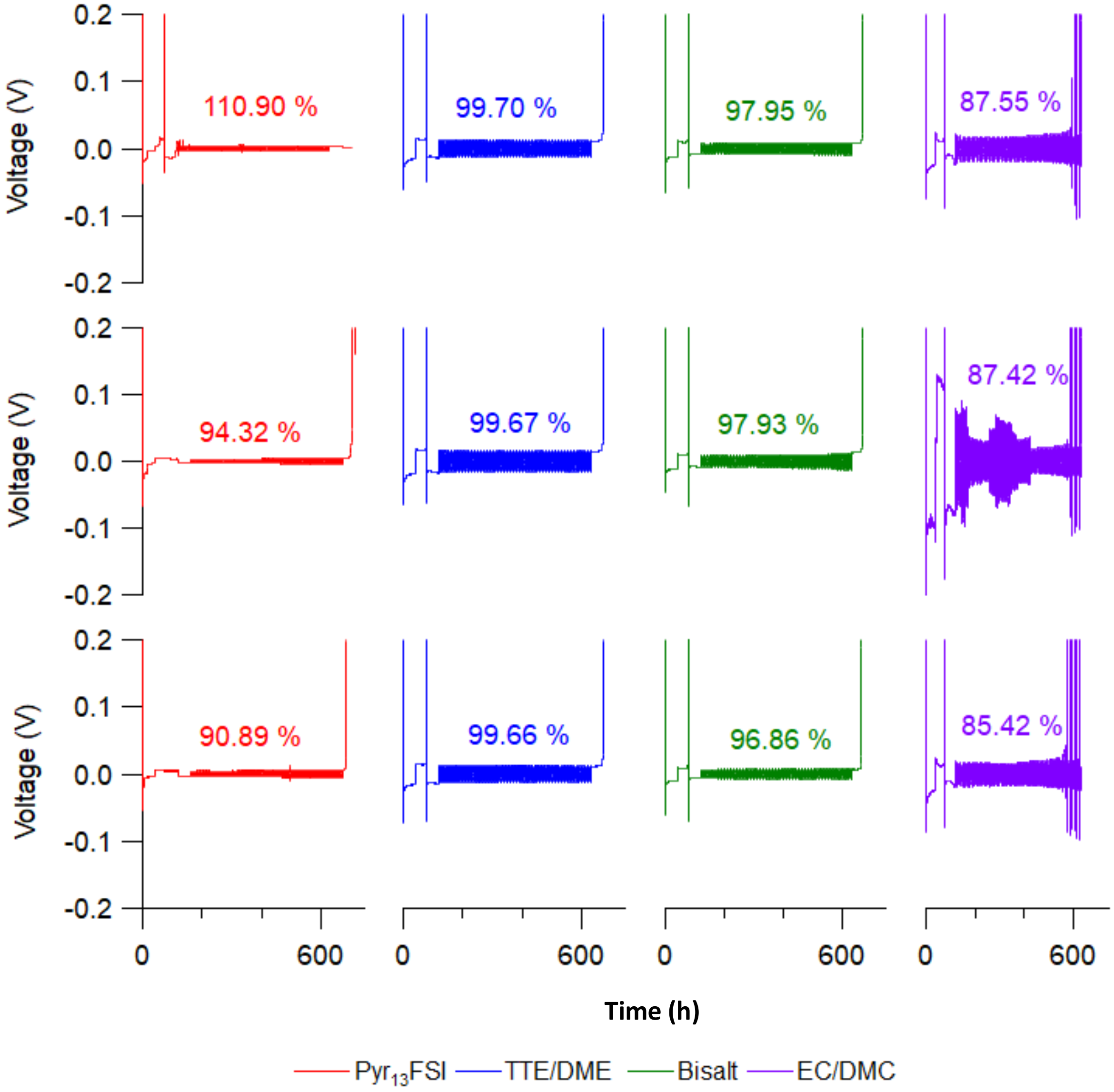

Figure S7: Chronopotentiometric determination of the Coulombic efficiency of Li plating/stripping in three separate $Pyr_{13}FSI$, TTE/DME, bisalt, and EC/DMC cells. TTE/DME and bisalt cells show high (>95%) CE and uniform stripping/plating behavior, while the EC/DMC and $Pyr_{13}FSI$ cells show inefficient stripping/plating behavior and parasitic reactions that reduce Coulombic efficiency. $Pyr_{13}FSI$ cells used 750 µm Li whereas all other cells used 50 µm Li

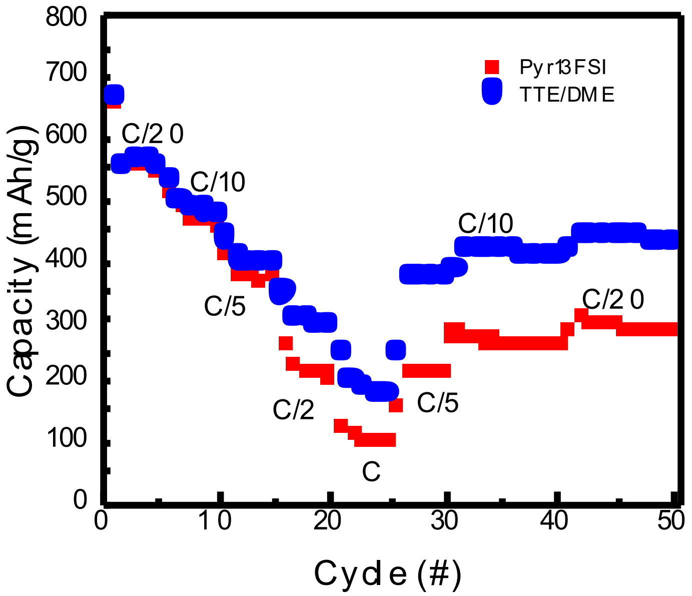

Figure S8: Rate-cycling comparison of $Pyr_{13}FSI$ and TTE/DME cells versus 750 µm Li foil (n = 1). TTE/DME is able to cycle with higher capacity at faster rates (1C) than $Pyr_{13}FSI$ and recovers its initial capacity upon return to slower rates (C/20).

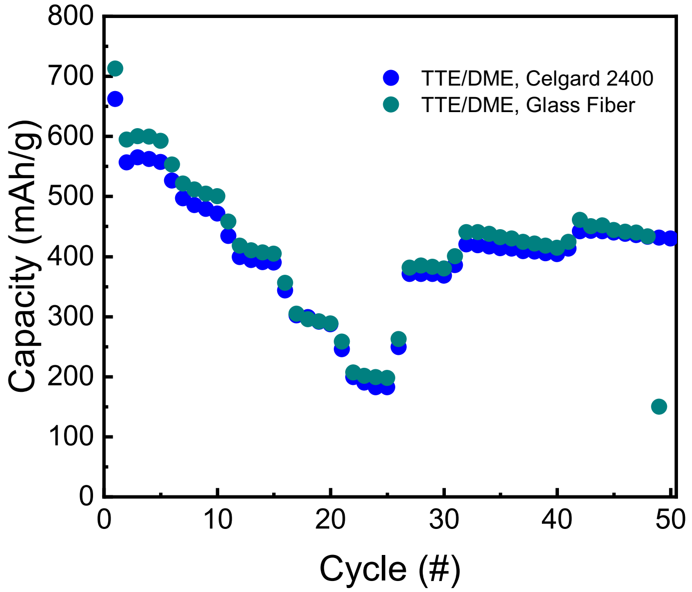

Figure S9: Rate-cycling comparison of TTE/DME cells versus 750 μm Li foil (n = 1) built using Celgard 2400 or glass fiber separators. The similar performance of the two cells indicates that the separator has minimal impact on cell capacity and rate-performance.

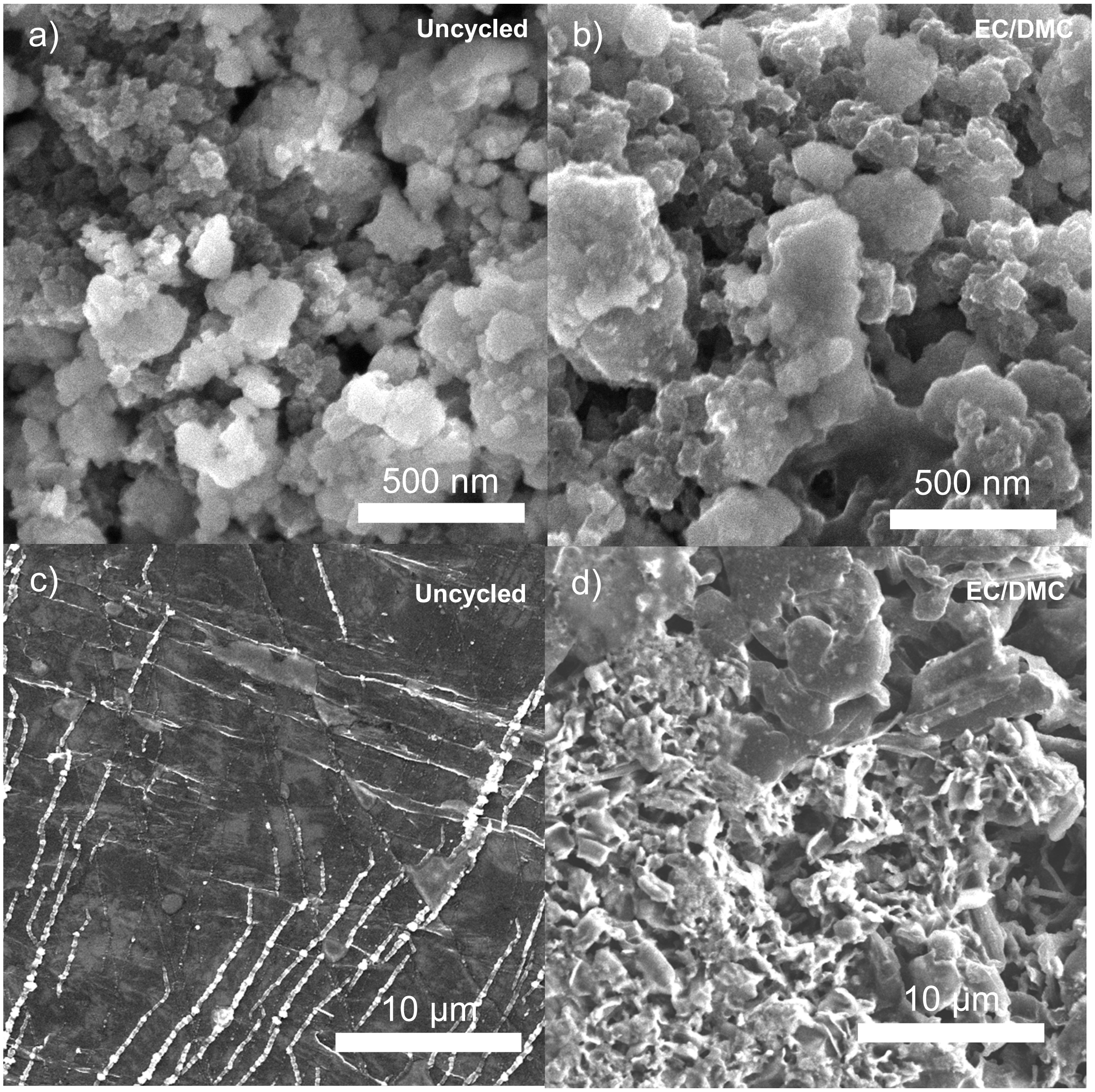

Figure S10 : SEM images of FeF_3/C cathodes a) prior to cycling and b) after cycling in EC/DMC for two cycles, showing minimal change to microscale morphology. c) Li anode prior to cycling and d) after being cycled twice in EC/DMC. The white lines composed of small nodules on c) indicate exposure to air and the formation of Li_2O. The Li deposits in d) appear porous and high surface area.

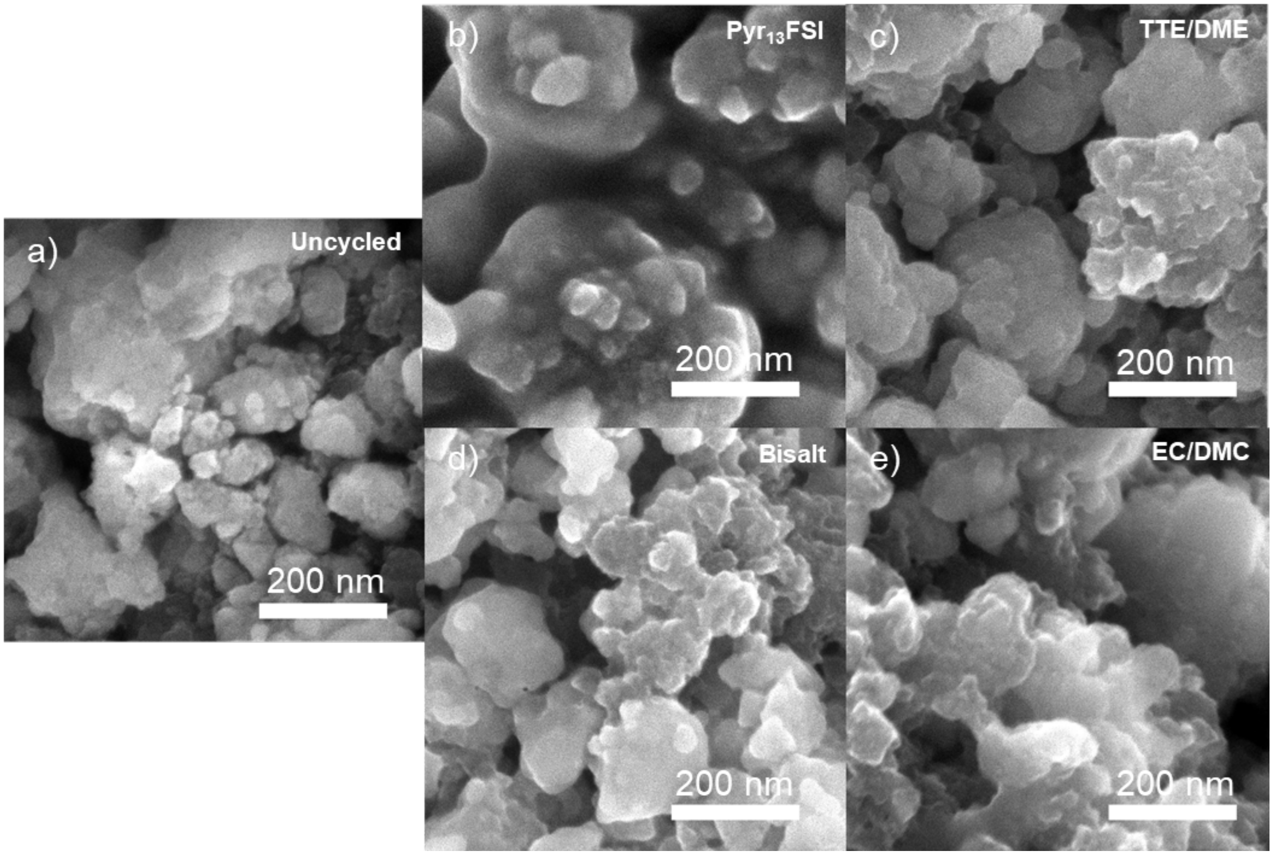

Figure S11: SEM images of a) an uncycled FeF_3/C cathode and cathodes cycled twice in b) $Pyr_{13}FSI$, c) TTE/DME, d) bisalt, and e) EC/DMC. The $Pyr_{13}FSI$ sample shows a thick surface layer of CEI/adsorbed ionic liquid on the surface of the cathode, while all other samples show clearly defined particles of FeF_3/C with no obvious differences after cycling.

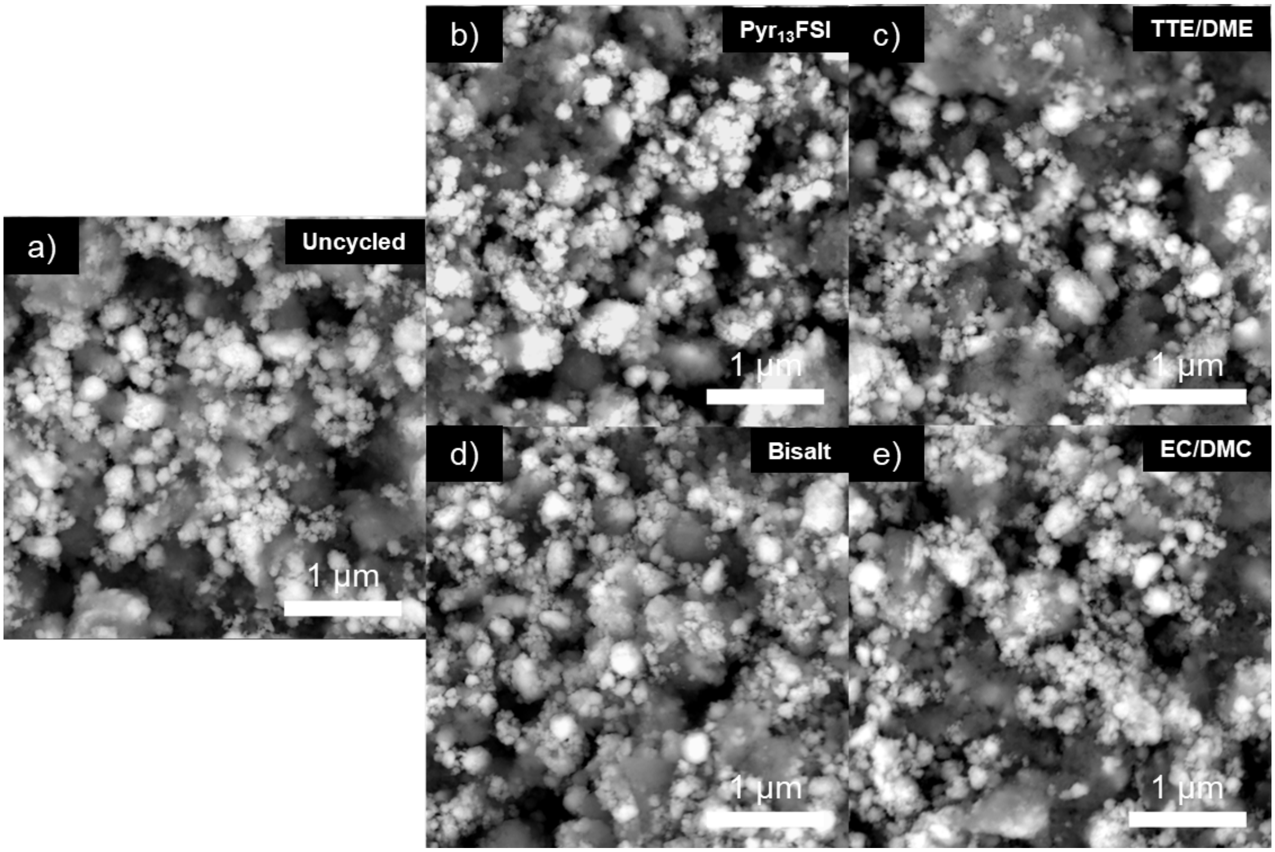

Figure S12: Electron backscatter SEM images of a) an uncycled FeF_3/C cathode and cathodes cycled two cycles in b) $Pyr_{13}FSI$, c) TTE/DME, d) bisalt, and e) EC/DMC. All images show less than 500 nm agglomerates of FeF_3 (lighter contrast) particles mixed homogeneously with carbon (darker contrast) particles.

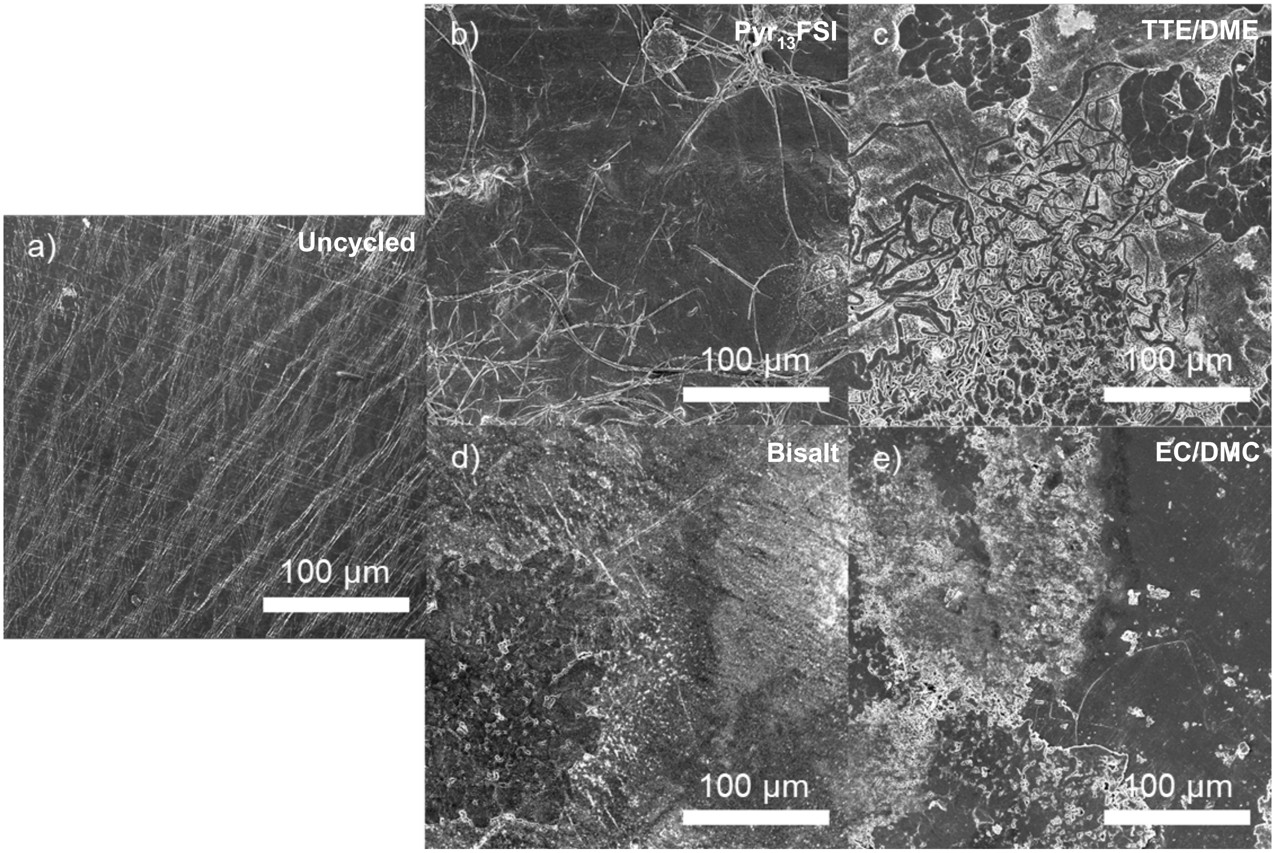

Figure S13: SEM images of Li anodes a) prior to cycling and after being cycled twice in b) $Pyr_{13}FSI$, c) TTE/DME, d) bisalt, and e) EC/DMC. All samples show evidence of slight O_2 exposure during transfer to the SEM. TTE/DME and bisalt anodes shows extended low-surface area deposits of Li, while the EC/DMC shows much higher surface area deposits. The $Pyr_{13}FSI$ cycled anode shows no clear Li deposition in areas uncoated by glass fiber, though Li may be hidden underneath the fiber in other areas.

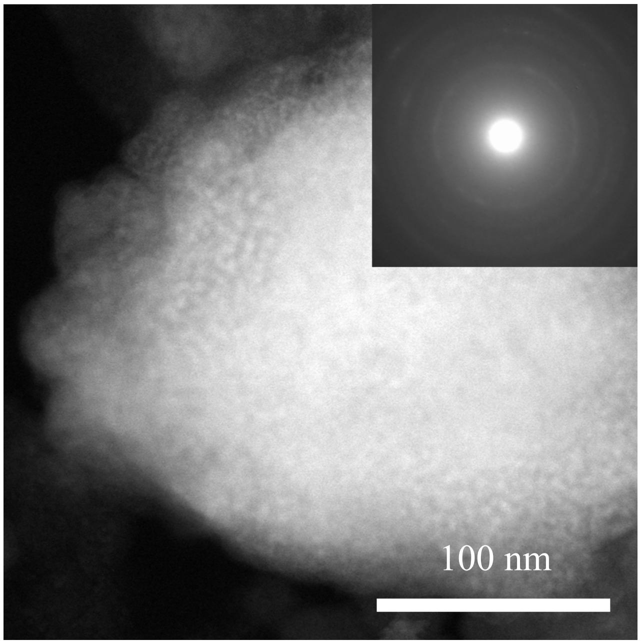

Figure S14: STEM images of an FeF_3 cathode cycled in EC/DMC electrolyte for 2 cycles with corresponding SAED pattern (upper right). The roughly 200 nm diameter secondary particle is composed of smaller 5 nm particles in an amorphous matrix, indicated by the broadness of the SAED spots in the insert.

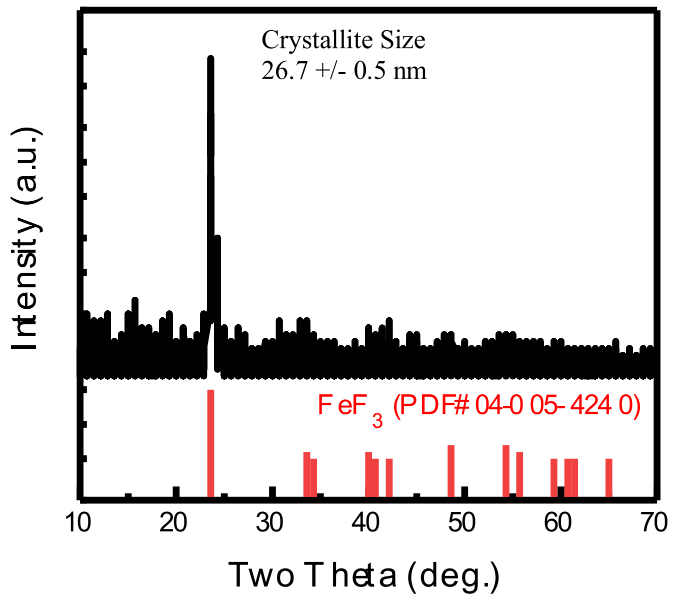

Figure S15: XRD spectra of FeF_3/C composite after ball milling before any cycling.

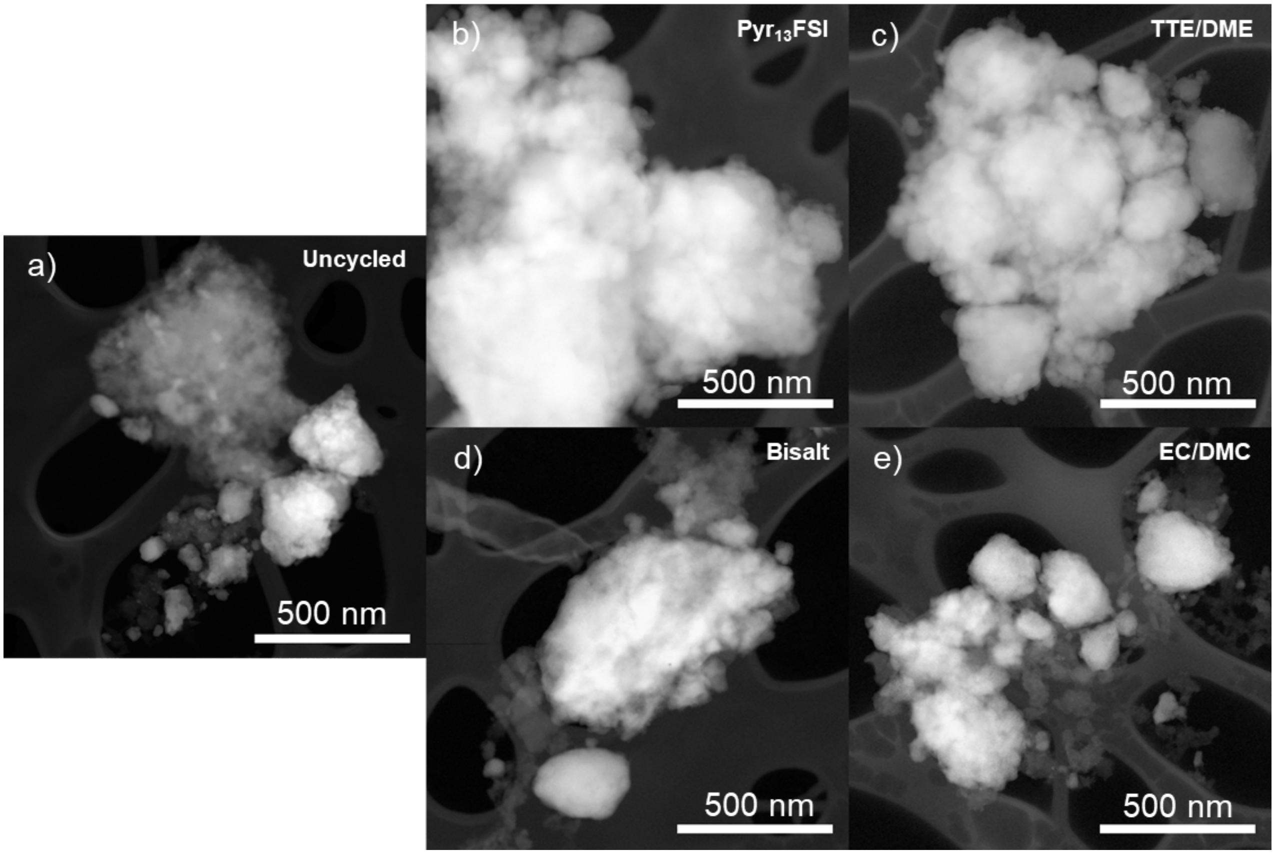

Figure S16: STEM images of a fresh (a) FeF_3 cathode and FeF_3 cathodes cycled in b) $Pyr_{13}FSI$, c) TTE/DME, and d) Bisalt electrolyte for 2 cycles. The secondary agglomerated particles show little change after cycling and remain approximately 500 nm in diameter.

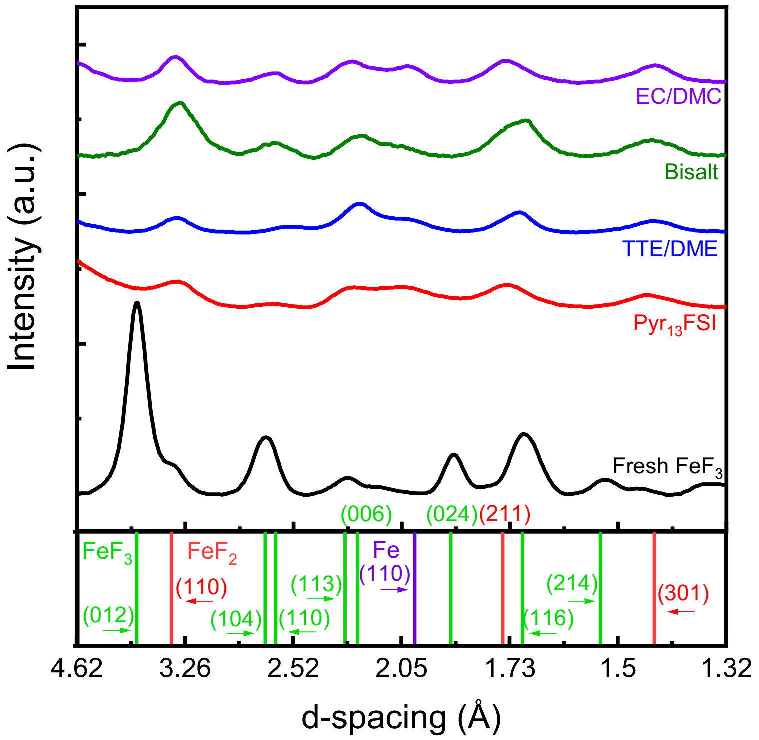

Figure S17: Radial distribution plot of SAED patterns from FeF_3 cathode material cycled 2 cycles in each electrolyte, integrated over 360° and indexed as FeF_3, FeF_2, and Fe.

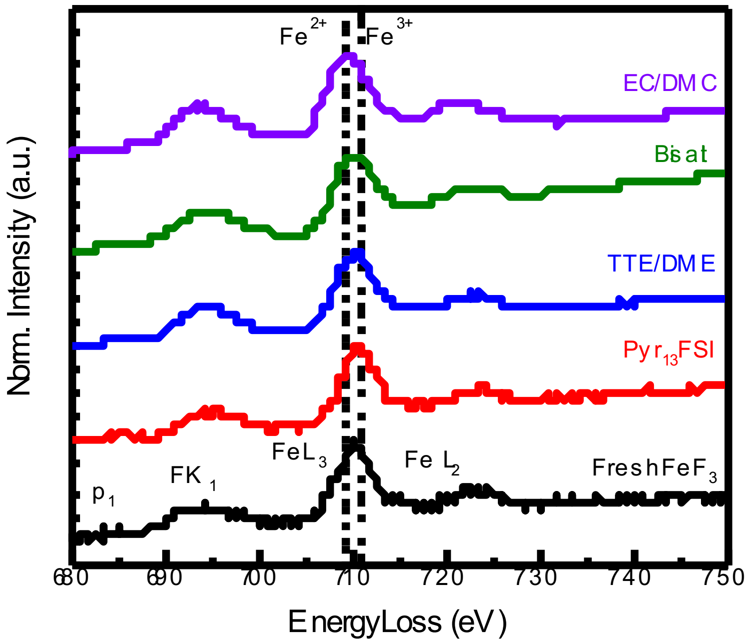

Figure S18: EELS spectra of the F K_1 and Fe L_3/L_2 region for FeF_3 cathode samples cycled for two cycles. The dashed line near 711 eV corresponds to Fe^{3+} character, while the dotted line near 709 eV corresponds to Fe^{2+} character.

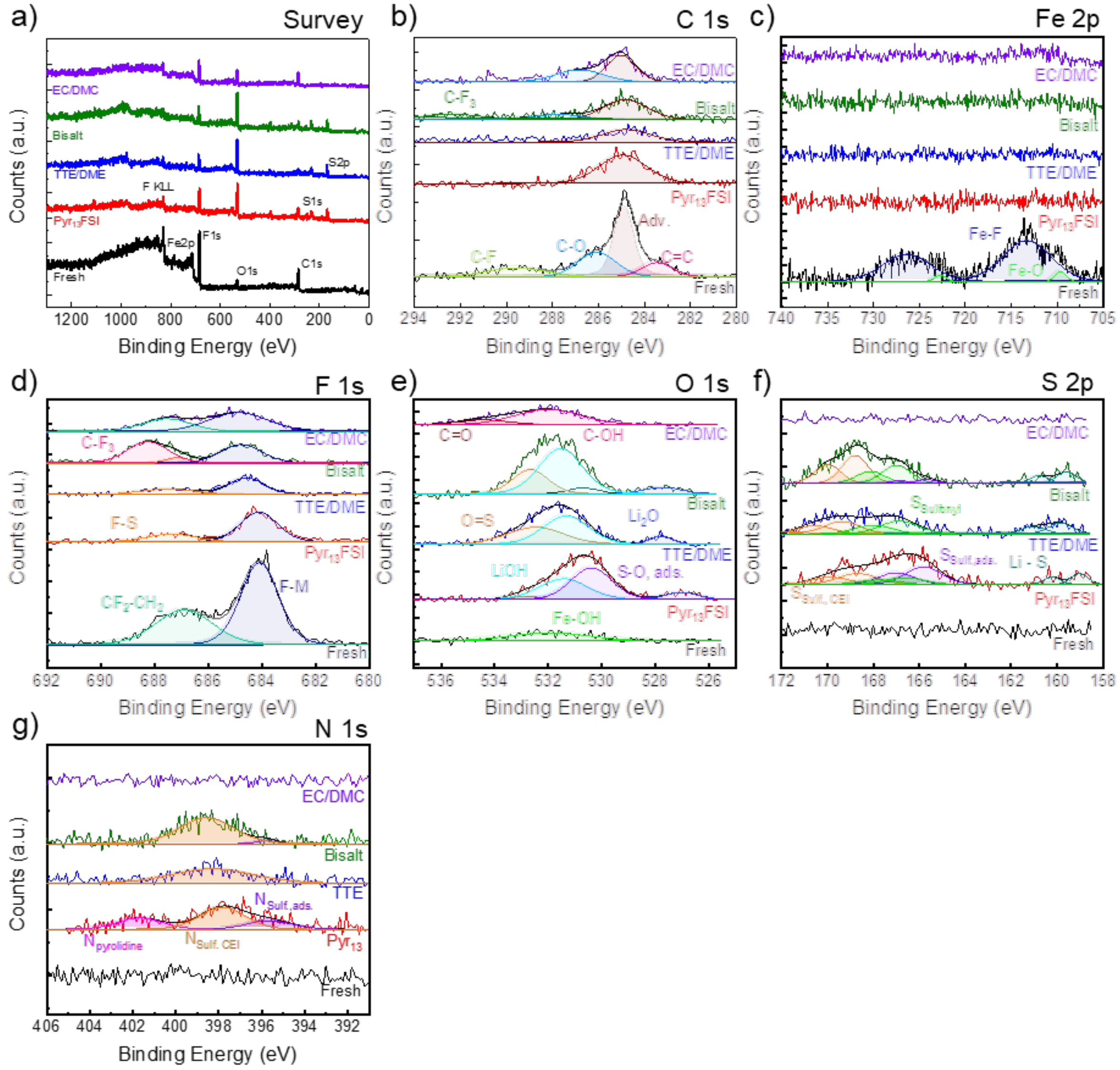

Figure S19: a) XPS survey spectra and spectra from the b) C1s, c) Fe 2p, d) F1s, e) O1s, f) S 2p, and g) N 1s regions of fresh and tested (two cycles) FeF_3 cathodes with assigned peaks.

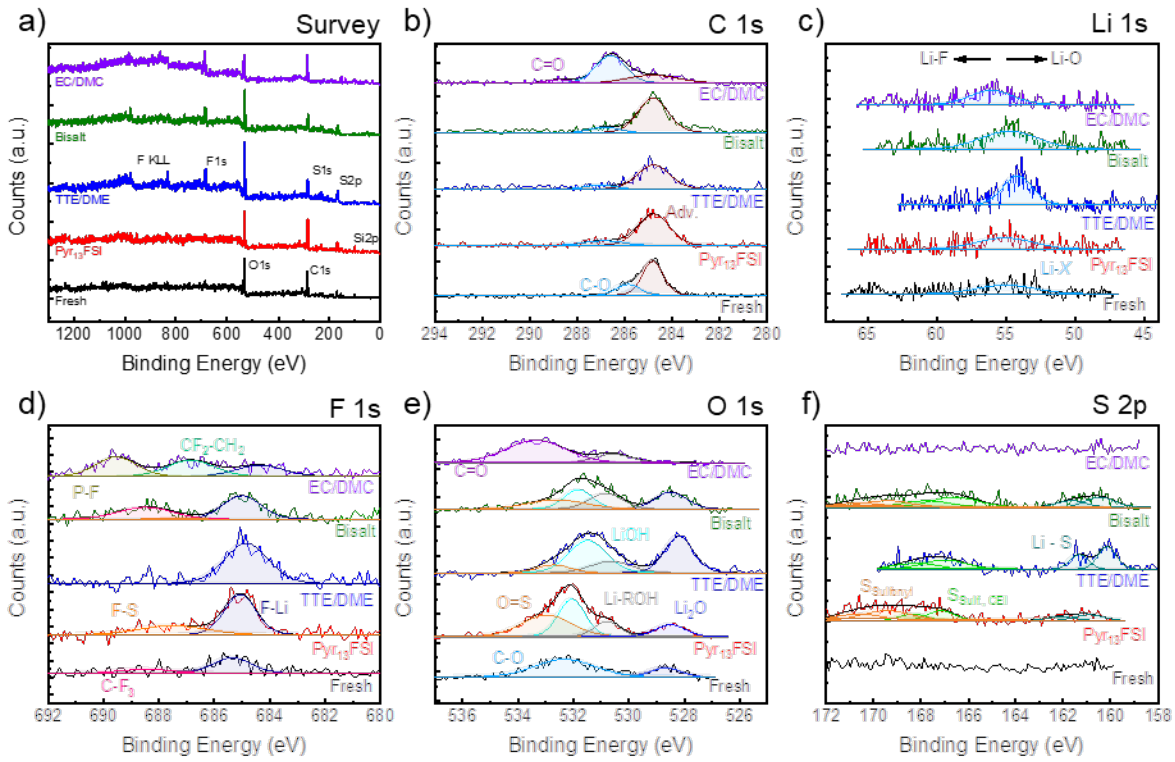

Figure S20: a) XPS survey spectra and spectra from the b) C1s, c) Li 1s, d) F1s, e) O1s, and f) S 2p regions of fresh and tested Li anodes with assigned peaks.

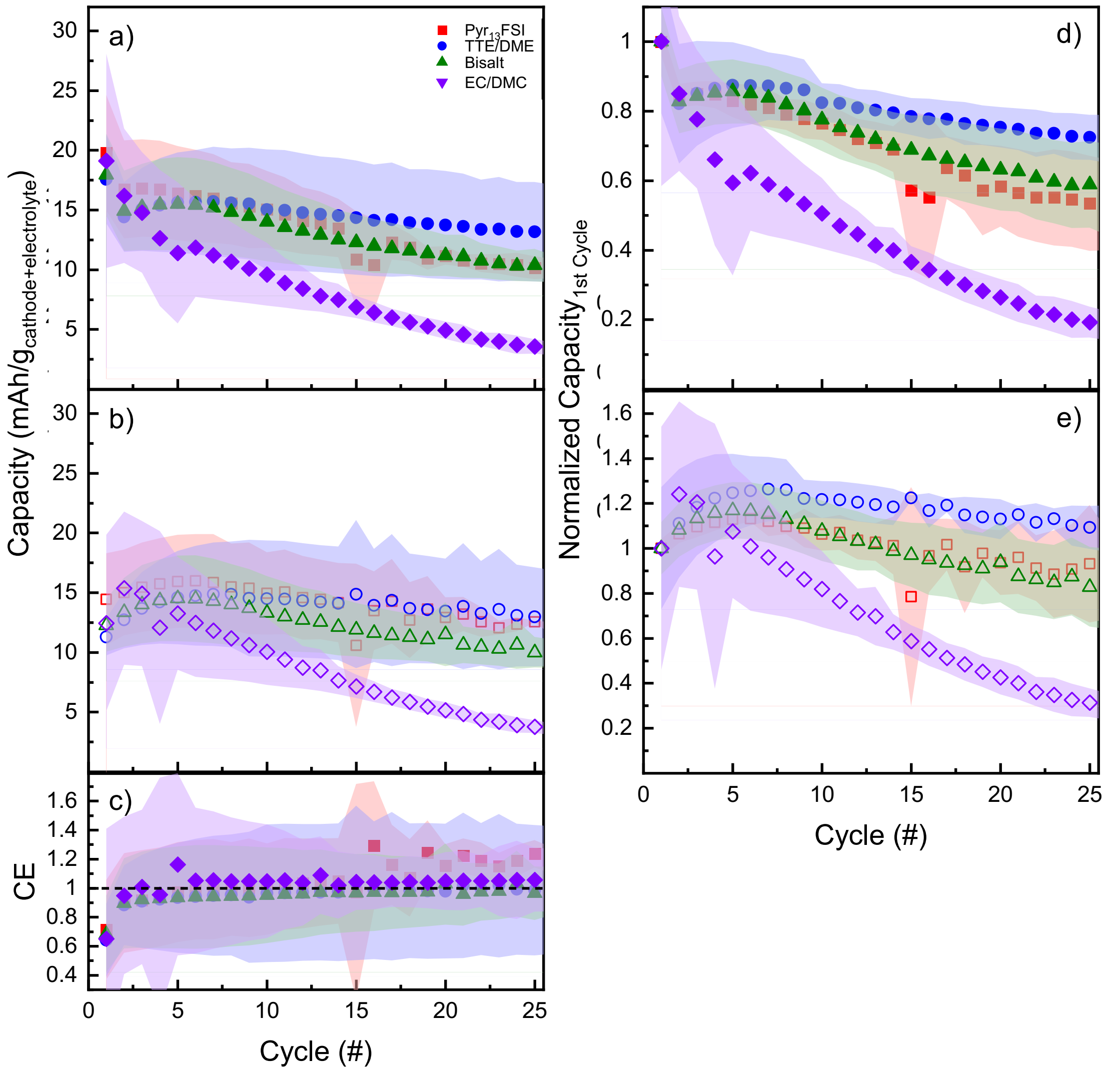

Figure S21: Average a) discharge and b) charge capacity of FeF_3/Li cells, tested using limited-Li metal anodes (20 µm) in $Pyr_{13}FSI$, TTE/DME, bisalt, and EC/DMC electrolytes at C/20 and reported per gram of cathode and electrolyte mass. c) Coulombic efficiency of limited-Li metal cells shown in a) and b). Average d) discharge and e) charge capacity of cells in a), normalized to capacity of first cycle to highlight difference in stability. Averages and standard deviation (shaded areas) are calculated from three cells (n = 3) and reported as mean±SD.

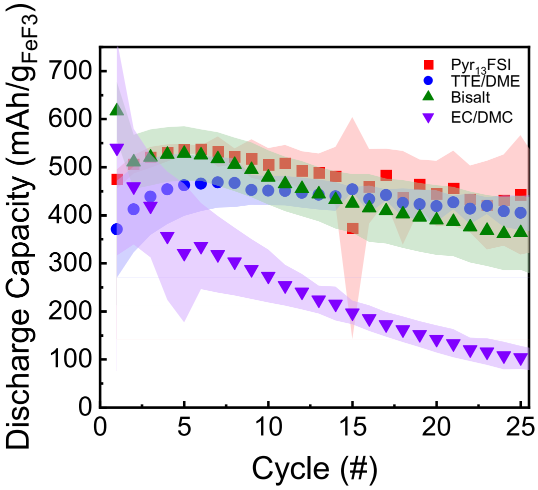

Figure S22: Average discharge of FeF_3/Li cells, tested using limited-Li metal anodes (20 µm) in Pyr_{13}FSI, TTE/DME, bisalt, and EC/DMC electrolytes at C/20. Capacity is normalized per gram of FeF_3 in the cathodes. Averages and standard deviation (shaded areas) are calculated from three cells (n = 3) and reported as mean±SD.

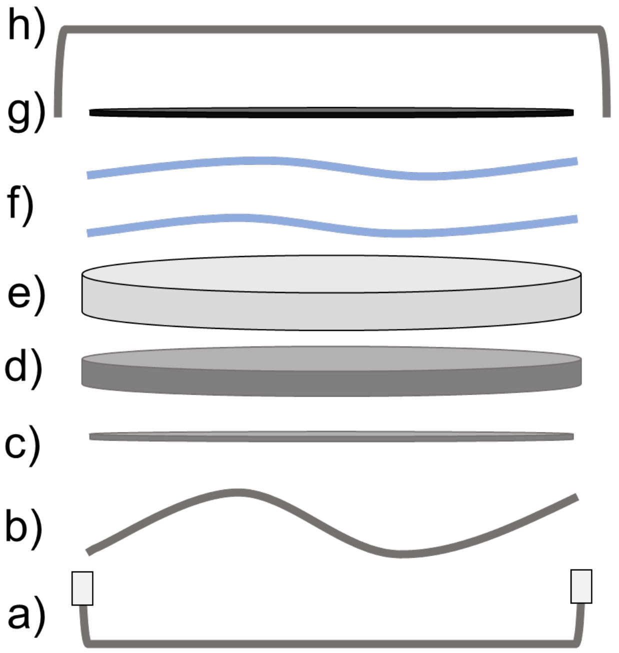

Figure S23: Schematic of a typical coin cell. a) stainless steel anode cap, b) stainless steel wave spring, c) 0.1 mm thick x 15.5 mm diameter stainless steel spacer, d) 0.5 mm thick x 16 mm diameter stainless steel spacer, e) 0.75 mm thick x 16 mm diameter Li foil, f) 19 mm diameter separators, g) 16 mm diameter FeF_3/C/PVDF cathode on C-coated Al foil, h) stainless steel cathode cap. Two Celgard 2400 separators are used to build the TTE/DME and bisalt electrolyte cells, two W-SCOPE separators are used for the EC/DMC electrolyte, and one Whatman glass microfiber (GF/C) separator was used with $Pyr_{13}FSI$ electrolyte. 70 µL of electrolyte was used for the TTE/DME, bisalt, and EC/DMC electrolytes, while 100 µL of the $Pyr_{13}FSI$ electrolyte was used.
